# Radiation with reticulation marks the origin of a major malaria vector

**DOI:** 10.1073/pnas.2018142117

**Published:** 2020-12-01

**Authors:** Scott T. Small, Frédéric Labbé, Neil F. Lobo, Lizette L. Koekemoer, Chadwick H. Sikaala, Daniel E. Neafsey, Matthew W. Hahn, Michael C. Fontaine, Nora J. Besansky

**Affiliations:** ^a^Department of Biological Sciences, University of Notre Dame, South Bend, IN 46556;; ^b^Eck Institute for Global Health, University of Notre Dame, South Bend, IN, 46556;; ^c^Wits Research Institute for Malaria, School of Pathology, Faculty of Health Sciences, University of the Witwatersrand, Johannesburg, Wits 2050, South Africa;; ^d^The Centre for Emerging, Zoonotic and Parasitic Diseases, National Institute for Communicable Diseases, Johannesburg 2131, South Africa;; ^e^National Malaria Elimination Centre, Chainama Hills Hospital Grounds, Lusaka, Zambia;; ^f^Department of Immunology and Infectious Diseases, Harvard TH Chan School of Public Health, Boston, MA 02115;; ^g^Genomic Center for Infectious Diseases, Broad Institute of MIT and Harvard, Cambridge, MA 02142;; ^h^Department of Biology, Indiana University, Bloomington, IN 47405;; ^i^Department of Computer Science, Indiana University, Bloomington, IN 47405;; ^j^Laboratoire MIVEGEC (Université de Montpellier, CNRS 5290, IRD 229), Centre de Recherche en Écologie et Évolution de la Santé (CREES), Institut de Recherche pour le Développement (IRD), F-34394, Montpellier, France;; ^k^Groningen Institute for Evolutionary Life Sciences (GELIFES), University of Groningen, Nijenborgh 7, 9747 AG Groningen, The Netherlands

**Keywords:** adaptive radiation, *Anopheles funestus*, anopheline mosquito species complex, introgression, reticulate evolution

## Abstract

Advances in genomics have led to an appreciation that introgression is common, but its evolutionary consequences are poorly understood. In recent species radiations the sharing of genetic variation across porous species boundaries can facilitate adaptation to new environments and generate novel phenotypes, which may contribute to further diversification. Most *Anopheles* mosquito species that are of major importance as human malaria vectors have evolved within recent and rapid radiations of largely nonvector species. Here, we focus on one of the most medically important yet understudied anopheline radiations, the Afrotropical *Anopheles funestus* complex (AFC), to investigate the role of introgression in its diversification and the possible link between introgression and vector potential. The AFC comprises at least seven morphologically similar species, yet only *An. funestus sensu stricto* is a highly efficient malaria vector with a pan-African distribution. Based on de novo genome assemblies and additional whole-genome resequencing, we use phylogenomic and population genomic analyses to establish species relationships. We show that extensive interspecific gene flow involving multiple species pairs has shaped the evolutionary history of the AFC since its diversification. The most recent introgression event involved a massive and asymmetrical movement of genes from a distantly related AFC lineage into *An. funestus*, an event that predated and plausibly facilitated its subsequent dramatic geographic range expansion across most of tropical Africa. We propose that introgression may be a common mechanism facilitating adaptation to new environments and enhancing vectorial capacity in *Anopheles* mosquitoes.

## Significance

Introgressive hybridization is prevalent in recent and rapid animal radiations, and emerging evidence suggests that it leads to the sharing of genetic variation that can facilitate adaptation to new environments and generate novel phenotypes. Here we study a recent and rapid radiation of African mosquitoes in which only one species, *An. funestus*, is a primary human malaria vector with a continent-wide geographic distribution. We trace the evolutionary history of the group and demonstrate introgression events between multiple species, the most recent of which involved substantial gene flow into *An. funestus* that preceded its range expansion across tropical Africa. Our findings point to introgression as an underappreciated factor contributing to the acquisition of high malaria vectorial capacity.

Once considered a rare anthropogenic aberration in animals, interspecific hybridization is now recognized to be both taxonomically widespread and pervasive, particularly in rapidly diversifying groups (1–3). Moreover, mounting genome-scale evidence suggests that introgression, the genetic exchange between species through hybridization and backcrossing, is also prevalent and may be consequential for evolution. Examples from fish, birds, mammals, and insects—including *Anopheles* mosquitoes—have shown that introgressed variation favored by natural selection can facilitate adaptation, enhance fitness, and drive evolutionary innovation and diversification (4–7). It has been postulated that introgressive hybridization is most prevalent in species-rich and rapidly diversifying radiations (2, 3, 8). Introgression in these groups may solely be opportunistic, given the multiplicity of young species in geographic proximity, but the process may also favor adaptive radiation through the generation of completely novel phenotypes (6, 9, 10).

There are three to four dozen *Anopheles* mosquito species that are of major importance as human malaria vectors, and all have evolved within recent and rapid radiations of morphologically cryptic species (informally classified as species complexes) (11, 12). Most members of these species complexes play no or very minor roles in disease transmission. The repeated de novo origin of major malaria vectors across these independent species radiations therefore holds clues about the nature of key evolutionary innovations that confer the ability to transmit disease widely and efficiently. However, most *Anopheles* species complexes are understudied. This is especially true of the secondary or nonvector species for which genomic resources are lacking, and basic knowledge of distribution, ecology, and behavior is scant.

Until now, the single best-studied group has been the *Anopheles gambiae* complex, composed of at least eight morphologically indistinguishable species that diversified rapidly and recently, likely within the last half-million years (7, 13, 14). Phylogenomic analysis revealed widespread genealogical discordance (7). Some discordance was due to incomplete lineage sorting as a result of both rapid radiation and large effective population sizes (7), but the majority was caused by massive introgression between the main vector species, involving both the autosomes and the centromere-proximal region of the X chromosome. So extensive was its impact that the inferred species branching order was evident in only 2% of the genome—mostly on the distal portion of the X chromosome, which is protected from introgression by a succession of fixed chromosomal inversion differences.

**Fig. 1.**
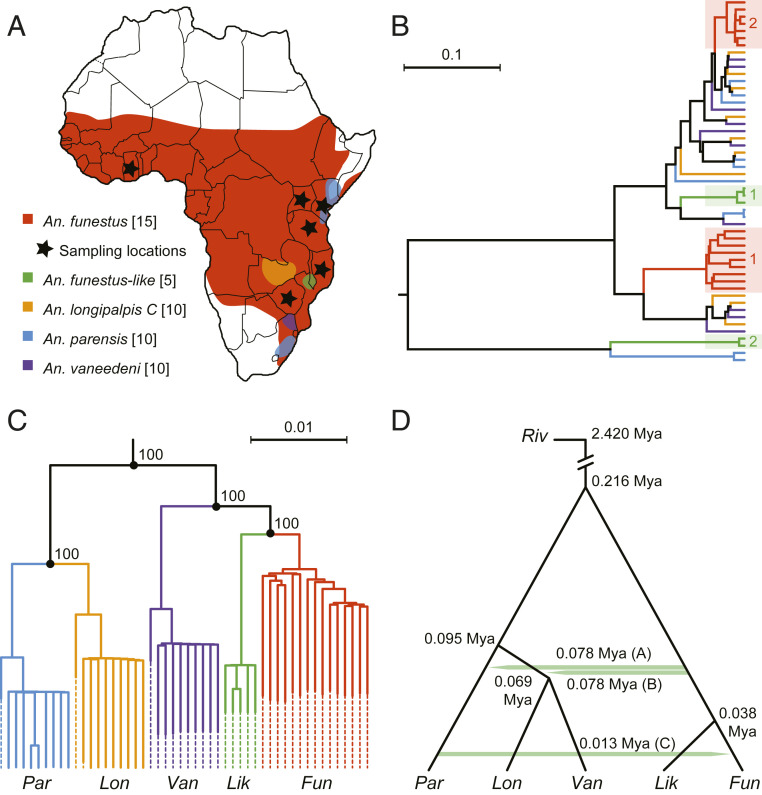
Distribution and genetic variation in the AFC. Color coding of species is consistent across panels. (*A*) Location and distribution of sampled species, adapted from ref. 21. Approximate sample locations for *An. funestus* are indicated by a black star. For full sample information, see *SI Appendix*, Table S1. (*B*) Phylogeny of complete mtDNA genomes constructed using BEAST2 indicating divergent clades of *An. funestus* (red shading) and *An. funestus-like* (green shading) (see *SI Appendix*, Fig. S12 for phylogeny with outgroup). (*C*) Neighbor-joining phylogeny averaged over the complete nuclear genome. (*D*) Summary evolutionary history displaying three introgression events as inferred by the methods described in the main text. Introgression events shown as green horizontal arrows between pairs of species indicate the majority direction of introgression. Median divergence and introgression times are displayed in millions of years ago (Mya). See *SI Appendix*, Table S11 for details. *An. funestus* (Fun), *An. funestus-like* (Lik), *An. longipalpis C* (Lon), *An. parensis* (Par), and *An. vaneedeni* (Van), *An. rivulorum* (Riv).

One of the most medically important of the understudied *Anopheles* species complexes is the Afrotropical *Anopheles funestus* complex (AFC). The AFC comprises at least seven morphologically similar species (15–18), yet only *An. funestus sensu stricto* (hereafter, *An. funestus*) is a highly efficient malaria vector, rivaled in importance solely by *An. gambiae* and its sister species *Anopheles coluzzii* in the *An. gambiae* complex (19–22). Comparative genomics of these two complexes may therefore be instructive with regard to malaria vectorial capacity. Both groups diversified in sub-Saharan Africa and may have experienced common geographic, ecoclimatic, and anthropogenic forces that shaped their history. In addition, the primary vector *An. funestus* broadly shares several characteristics with primary vectors in the *An. gambiae* complex: a geographic range that encompasses most of tropical Africa (Fig. 1*A*), high levels of chromosomal inversion polymorphism (23–25), large effective population size, and little population genetic structure across the continent (26, 27). Furthermore, the discovery of two very distantly related mitochondrial DNA (mtDNA) haplotypes (clades 1 and 2) segregating in *An. funestus* (27) raises the prospect of historical introgression analogous to that documented for *An. gambiae*, prompting an intriguing question: Can introgression be a source of evolutionary novelty leading to augmented vectoral capacity?

Here, we examine the role of introgression in the evolution of the AFC, using recent methods of phylogenetic network reconstruction that allow for divergence and reticulation to be inferred jointly. We use a combination of phylogenomic and population genomic analyses, based on de novo genome assemblies and additional whole genome resequencing, to: 1) establish species relationships, 2) determine the direction, extent, and genomic architecture of introgression across the complex, and 3) assess the role of introgression in the evolution of the primary vector *An. funestus*. We show that extensive interspecific gene flow involving multiple species pairs has shaped the evolutionary history of the AFC since its diversification ∼216 thousand years ago (Kya). The most recent introgression event ∼13 Kya involved a massive and asymmetrical movement of genes from a distantly related AFC lineage into *An. funestus*, an event that predated and plausibly facilitated its subsequent dramatic geographic range expansion across most of tropical Africa. We propose that introgression may be a common mechanism facilitating adaptation to new environments and enhancing vectorial capacity in *Anopheles* mosquitoes.

## Results

### Genome Sequencing and Assembly.

As a foundation for our analyses, we generated reference-assisted de novo genome assemblies (Table 1) from individual field-collected mosquitoes (Fig. 1*A* and *SI Appendix*, Texts S1–S3, Fig. S1, and Table S1). Augmenting the existing *An. funestus* AfunF3 reference from the FUMOZ colony (28), we assembled four new reference genomes from additional AFC species (*An. funestus-like*, *Anopheles parensis*, *Anopheles vaneedeni*, and *Anopheles longipalpis type C—*hereafter, *An. longipalpis C*). The only other AFC members, *Anopheles confusus* and *Anopheles aruni,* could not be obtained. Our de novo assemblies also included two outgroups: *Anopheles rivulorum* and *Anopheles species A*, the latter a previously recognized but formally undescribed species morphologically similar to *An. funestus* but distinctive in ITS2 sequence (>6% divergent from *An. funestus*; refs. 29, 30). Reference assemblies were contiguous on the X and chromosome arm 3R for all AFC species, while moderately fragmented scaffolds characterized chromosome arms 3L, 2R, and to a lesser extent, 2L (*SI Appendix*, Figs. S2 and S3 and Tables S2 and S3).

**Table 1.** Genome assembly statistics

Species	Country	Contigs	Size	N50	Scafs*	Size	BUSCO^†^				Accession
							Single	Duplicate	Fragmented	Missing	
*An. funestus-like*	Malawi	15,489	209,420,710	67,275	9	201,162,353	95.5%	2.6%	1.1%	0.8%	STHE00000000
*An. longipalpis C*	Zambia	33,338	323,670,219	30,221	7	220,976,958	75.1%	18.2%	3.3%	3.4%	STHD00000000
*An. parensis*	South Africa	26,828	251,769,316	50,161	10	216,971,959	87.3%	9.2%	1.9%	1.6%	STHC00000000
*An. vaneedeni*	South Africa	27,582	279,105,143	46,024	8	225,734,782	82.7%	13.9%	2.7%	0.7%	STHA00000000
*An. species A*	Kenya	21,640	242,997,558	74,383	15	192,881,626	98.0%	1.0%	0.5%	0.5%	STHF00000000
*An. rivulorum*	South Africa	37,847	273,938,921	26,085	14	199,718,359	92.3%	4.6%	1.7%	1.4%	STHB00000000

* Reference-assisted scaffolding with ragout and AfunF3.

^†^ Percent calculated out of 1,066 total BUSCOs.

In support of population genomic analyses and simulations, we also individually resequenced the genomes of 42 field-collected mosquitoes representing five AFC species (*SI Appendix*, Table S1). These included eight specimens each of *An. longipalpis C*, *An. parensis*, and *An. vaneedeni*; three of *An. funestus-like*; and 15 of *An. funestus*, six of which carried clade 2 mtDNA.

### Resolving the Species Tree Despite a Complex History of Introgression.

Species relationships in the AFC have not been confidently resolved. Previous efforts to reconstruct phylogenies using fragments of two mtDNA genes uncovered widespread paraphyly (31), a pattern that we confirm here based on complete mtDNA genome sequences (Fig. 1*B* and *SI Appendix*, Fig. S12). A neighbor-joining tree averaged over the entire nuclear genome reveals reciprocal monophyly among species, while the mtDNA tree shows extensive paraphyly (Fig. 1 *B* and *C* and *SI Appendix*, Text S6). Moreover, *An. funestus* and *An. funestus-like* each contain two highly divergent mtDNA clades (Fig. 1*B* and *SI Appendix*, Fig. S12), consistent with the possibility of historical introgression events resulting in mitochondrial capture. Ribosomal DNA (rDNA) second internal transcribed spacer (ITS2) sequences, instrumental for taxonomic identification of morphologically cryptic species in the AFC (32, 33), also hint at possible historical introgression in this species group. Instead of the near-complete sequence identity expected among units of tandemly arrayed rDNA (34), we found that *An. longipalpis C* possesses two types of ITS2, one highly similar to *An. parensis* and the other highly similar to *An. vaneedeni* (*SI Appendix*, Fig. S1), in agreement with previous findings (15).

**Fig. 2.**
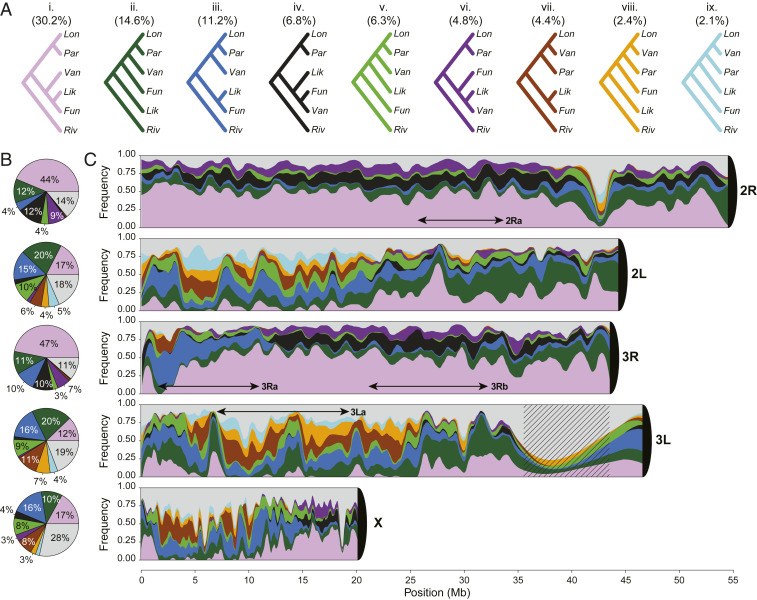
Frequency and distribution of gene trees. Phylogenetic trees were reconstructed in 5 kb nonoverlapping windows along the chromosomes using PhyML. Color coding of topologies is consistent across panels. (*A*) Nine major topologies (i–ix) found on any chromosome arm with a frequency of at least 5%. Normalized whole-genome frequencies are indicated in parentheses. (*B*) The frequency of each major topology on individual chromosome arms. Less frequent topologies are pooled together and displayed in gray in *B* and *C*. (*C*) Chromosome painting representing the frequency of topologies across chromosome arms. For display purposes the frequencies are averaged across adjacent windows. Approximate locations of common chromosomal inversions in *An. funestus* (3Ra, 3Rb, 3La, and 2Ra) are indicated by double-headed arrows. Centromeres are represented as black 1/4 circles. Hatching represents a masked region. *An. funestus* (Fun), *An. funestus-like* (Lik), *An. longipalpis C *(Lon), *An. parensis *(Par), *An. vaneedeni *(Van), and *An. rivulorum* (Riv).

To explore phylogenetic relationships in the AFC, we used nonoverlapping windows 5 kb in length from the full five-species nuclear genome alignment (plus two outgroups; *SI Appendix*, Texts S4 and S6). Excluding masked heterochromatic, repetitive regions and windows not passing quality filters, this resulted in 24,556 windows spanning ∼123 Mb of aligned sequence. Reconstructing maximum likelihood phylogenies from each of these windows, we observed all possible topologies (*n* = 105) at least once. The most common topology (denoted tree **i**) was present at more than twice the frequency of the next most-common tree, having a genome-wide frequency of ∼30% (Fig. 2*A* and *SI Appendix*, Fig. S13). This topology is found in highest proportion across most of the length of chromosome arms 2R (44%) and 3R (47%) (Fig. 2 *B* and *C*). Its distribution is much more restricted on 2L, 3L, and especially the X chromosome where it is largely absent from positions ∼1.6 to ∼6.8 Mb (Fig. 2 *B* and *C*).

Importantly, there are nine topologies observed frequently on at least one chromosome arm, reflecting substantial genealogical discordance (Fig. 2 and *SI Appendix*, Fig. S13). Their heterogeneous distribution along the genome appears idiosyncratic to individual chromosome arms rather than being driven by a common landscape of reduced recombination near centromeres or telomeres (Fig. 2*C*). Arm-specific topological patterns are not obviously related to the location of common chromosomal inversions known to segregate in *An. funestus* populations across tropical Africa (2Ra, 3Ra, 3Rb, and 3La; refs. 21, 24, 25) (Fig. 2*C*). Furthermore, in contrast to the pattern observed in the *An. gambiae* complex (7), we find no striking difference in the nature or frequency of autosomal versus X chromosome topologies; the most common trees on the autosomes (topologies **i**–**iii**) also are the most frequent on the X chromosome (*SI Appendix*, Fig. S14).

**Fig. 3.**
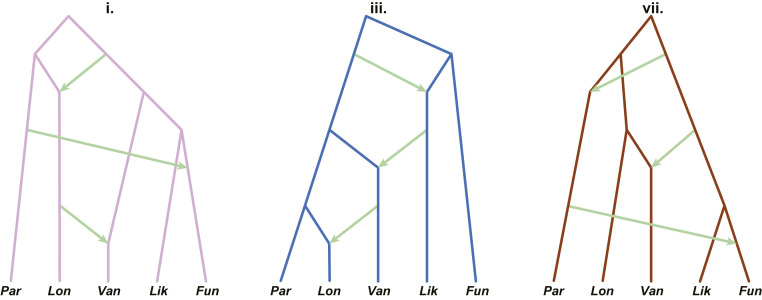
Competing models representing the evolutionary history of the AFC inferred from admixture graphs. Lineage divergences correspond to major topologies i, iii, and vii in Fig. 2. Lineage reticulations and the inferred direction are indicated by green arrows. Models are not scaled to time. *An. funestus* (Fun), *An. funestus-like *(Lik), *An. longipalpis C *(Lon), *An. parensis* (Par), and *An. vaneedeni *(Van).

Facing phylogenetic uncertainty owing to incomplete lineage sorting (ILS) and/or introgression, we sought to resolve the true bifurcation history of the AFC by adopting an approach that allows for both divergence and reticulation using *D*-statistics (35, 36) and admixture graphs to evaluate the fit of each history to the data (*SI Appendix*, Text S7). To implement this approach, *D*-statistics (which are robust to the presence of natural selection, ref. 37) were calculated for all AFC species triplets using *An. rivulorum* as an outgroup (*SI Appendix*, Fig. S15 and Table S6). Starting with the nine most frequent window topologies from the whole-genome analysis (Fig. 2*A*), we built admixture graphs for each, adding up to three reticulations in all possible arrangements, and chose the graph with the highest likelihood (lowest cost function) (*SI Appendix*, Fig. S16). Remaining models based on competing topologies were compared in pairs using a likelihood ratio test. Any model that could not be rejected in favor of another was retained as an equally likely representation of AFC evolutionary history. This left three models (Fig. 3) whose backbones reflect branching patterns observed among the most common bifurcating trees inferred from the whole-genome analysis (trees **i**, **iii**, and **vii**; Fig. 2) but with three reticulations each.

To identify the most likely evolutionary history among the three, we used approximate Bayesian computation (ABC) with a supervised machine learning model-selection procedure, a computationally tractable approach even for large datasets (38). For this analysis, we expanded our samples from a single reference genome for each species to multiple resequenced individuals from each AFC species (Fig. 1*A* and *SI Appendix*, Tables S1 and S4). Model selection was based on a random forest trained on data simulated under each competing model (*SI Appendix*, Text S8). Simulations drew from the observed empirical distribution of values of genome-wide recombination rates and nucleotide diversity and were initialized with demographic histories inferred from each species. Summary statistics were calculated using either the whole genome or the noncoding regions only, under the assumption that noncoding regions are less affected by selection; the inferred histories were robust to the choice of loci (*SI Appendix*, Texts S5 and S8). The best model, based on the backbone topology **vii** plus three reticulation events (Fig. 3), received an average of 600 out of 1,000 votes across the autosomes and a posterior probability of 0.68 (*SI Appendix*, Table S10). Model **vii** thus represents the species graph, our working hypothesis for the true species tree of the AFC and the reticulation events that punctuated its history. According to this hypothesis, *An. funestus*-*like* and *An. funestus* are sister taxa, as are *An. vaneedeni* and *An. longipalpis C*. *An. parensis* is sister to the latter clade. The backbone topology (**vii** in Fig. 2*A*) is represented by only ∼4% of the genome because the treelike history has been almost entirely overwritten by multiple introgression events involving multiple pairs of nonsister taxa.

### Recent Introgression into *An. funestus* Preceded Its Continent-Wide Range Expansion.

To derive estimates of the timing of lineage splitting and introgression events, we used ABC with simulations under model **vii** (*SI Appendix*, Text S9). Estimates are summarized in generation times (*SI Appendix*, Table S11) and years (Fig. 1*D*), the latter assuming a mutation rate of 2.8 × 10^−9^ (39) and 11 generations per year. Under these assumptions, the initial radiation of the AFC—the divergence of the ([Van + Lon]Par) clade from the (Fun + Lik) clade—occurred ∼216 Kya (95% CI, 213–222 Kya). Next was the split between *An. parensis* and the (Van + Lon) clade at ∼95 Kya (91–100 Kya). There followed two temporally indistinguishable introgression events (A and B in Fig. 1*D*) dating to ∼78 Kya (68–87 Kya), both involving the ancestor of the (Fun + Lik) clade. Events A and B (Fig. 1*D*) featured gene flow from that ancestor into *An. parensis* and the ancestor of the (Van + Lon) clade, respectively. These introgression events closely preceded or overlapped the splitting of this lineage into *An. vaneedeni* and *An. longipalpis C* at ∼69 Kya (62–79 Kya). The most recent species split, leading to the sister taxa *An. funestus-like* and *An. funestus,* occurred only ∼38 Kya (31–45 Kya).

We estimate that the third introgression event from *An. parensis* into *An. funestus* (labeled “C” in Fig. 1*D*), occurred considerably more recently, only ∼13 Kya (12–15 Kya). Two lines of evidence suggest that this introgression preceded the range expansion of *An. funestus* into its current continent-wide distribution across tropical Africa. First, we sequenced *An. funestus* genomes sampled from six geographic localities spanning West, East, and Southern Africa (*SI Appendix*, Tables S1 and S4). The median divergence time among *An. funestus* populations inferred from two different approaches was ∼1.18 Kya (0.29–7.00 Kya) and ∼0.945 Kya (0.019–2.2 Kya) (*SI Appendix*, Text S5 and Table S5), estimates that are not significantly different (Wilcoxon rank-sum test, *P* value = 0.47). Both dates are substantially younger than the estimated introgression from *An. parensis* into *An. funestus*. Second, we verified that each *An. funestus* population was equally distant from *An. parensis* using pairwise genetic distance (*d*_XY_; *SI Appendix*, Text S9). Pairwise distances were highly similar between each *An. funestus* population and *An. parensis* (*d*_XY_ average 0.026, standard deviation 0.0004), whether or not population samples came from localities where the species potentially co-occur, consistent with historical rather than contemporary localized gene flow.

### Introgression Involved Substantial Fractions of Autosomes and the X Chromosome.

We next analyzed the distribution and directionality of introgression along the genome between pairs of AFC species using a supervised machine learning framework in the software package FILET, developed for this purpose (40). An extra trees classifier was trained on data simulated under our model of AFC evolutionary history to identify 10 kb windows along the genome with a high probability of introgression (*SI Appendix*, Text S10). We tested all 10 pairwise combinations of species, not only the pairs implicated by *D*-statistics, as *D*-statistics cannot detect gene flow between sister lineages and may also lack power to detect minor gene flow events. Moreover, because gene flow was inferred between the lineages leading to *An. funestus* and *An. parensis* at two separate time periods (Fig. 1*D*, events A and C), we attempted to distinguish these events by training the classifier on simulated data under two exclusive scenarios, one that allowed migration at event A but barred it at event C and a second under the converse. In all cases, we retained only those windows classified as introgressed with ≥90% probability.

To corroborate our results from FILET, an independent test of introgression based on alternative evidence was also applied (*SI Appendix*, Text S11). This statistical test (QuIBL) employs the distribution of internal branch lengths of triplet topologies discordant with the species tree to distinguish between ILS and introgression (4).

**Fig. 4.**
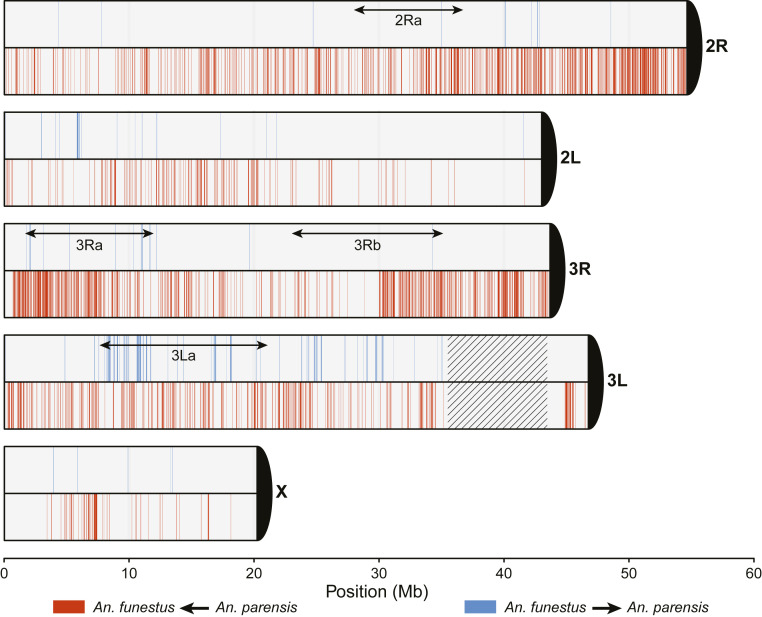
Genomic regions of introgression between *An. funestus* and *An. parensis*. Windows classified as introgressed between *An. funestus* and *An. parensis* with >90% probability are represented on each chromosome arm. Blue indicates introgression from *An. funestus* into *An. parensis*; red indicates introgression from *An. parensis* into *An. funestus*. Empty areas were classified either with lower confidence or as not introgressed. Approximate locations of common chromosomal inversions in *An. funestus* (3Ra, 3Rb, 3La, and 2Ra) are indicated by double-headed arrows. Centromeres are represented as black 1/4 circles. Hatching represents a masked region.

The results from both methods validate our inference of the three introgression events depicted in Fig. 1*D* (*SI Appendix*, Tables S13 and S14). Genomic regions predicted to be introgressed were heterogeneously distributed along the genome (Fig. 4 and *SI Appendix*, Figs. S18–S22). Furthermore, the directionality of gene flow was highly asymmetric (*SI Appendix*, Table S13). For example, we detected no introgression into *An. funestus* resulting from events A or B; the majority of introgression from these events was detected in the genomes of *An. parensis* and *An. vaneedeni* (at least 11 and 20 Mb, respectively; Fig. 1*D* and *SI Appendix*, Table S13). Notably, the most recent introgression event C was strongly biased in the direction of *An. funestus*, accounting for 31.6 Mb (22.5% of the accessible genome; *SI Appendix*, Table S13). FILET detected substantial introgression between some species pairs on the X chromosome as well as the autosomes (Fig. 4 and *SI Appendix*, Table S13). This was partly corroborated by QuIBL, but the power of this test to statistically distinguish ILS from introgression was limited on the X chromosome due to short branches and low counts (*SI Appendix*, Table S14).

We detected a fourth introgression event not uncovered in our earlier inferences of divergence and reticulation based on admixture graphs, as we had limited those analyses to only three reticulations. Both methods of detecting introgression applied here supported this event, which involved substantial X chromosome and autosomal gene flow mainly from *An. parensis* into *An. longipalpis C* (*SI Appendix*, Fig. S22; event D in *SI Appendix*, Tables S13 and S14). We also confirmed the absence of substantial introgression between sister taxa (events G and H, *SI Appendix*, Table S13), including *An. funestus* and *An. funestus-like*, which is particularly noteworthy in contrast to the prevalence of introgression between nonsister groups.

## Discussion

Africa bears >90% of the world’s burden of morbidity and mortality attributable to malaria principally because it is home to the most important *Anopheles* mosquito vectors. The most obvious attributes shared by these major vectors, which set them apart from their closely related minor or nonvector sibling species, are a high degree of anthropophily, a nearly pan-African species range, and abundant levels of both chromosomal inversion polymorphism and nucleotide diversity. In the historical literature there has been a supposition that these highly anthropophilic malaria vectors should be the most recently radiated members of species complexes, given their dependence on a resource—the human species—that was neither abundant nor widespread until relatively recently. For example, Coluzzi et al. (41) noted that “*A. gambiae* seems to be the least likely candidate for the ancestral line, as this highly anthropophilic species appears to be the product of a speciation process driven by human impact on the environment subsequent to the Neolithic revolution.” Yet when the elusive species tree for the *An. gambiae* complex was finally confidently resolved (7), this expectation did not necessarily fit the data: The lineage leading to the two most efficient vectors in the complex was one of the earliest to split (509 Kya according to the most recent estimate; ref. 13) not long after the initial radiation of the complex. Even in the AFC, where our data suggest that the divergence of *An. funestus* from *An. funestus-like* was indeed the most recent split, the divergence time of 38 Kya is not consistent with a human-influenced speciation process subsequent to the ∼5 Kya expansion of the Bantu-speaking agriculturalists from Central Africa across sub-Saharan Africa (42). More plausibly, the Bantu expansion could have helped to promote both the demographic and the geographic range expansions of both *An. funestus* and the primary vectors in the *An. gambiae* complex (43). What is striking in both species groups is the strongly asymmetric gene flow from nonsister taxa into the lineages that lead to major vectors, species whose invasive and synanthropic phenotypes seem to have emerged following hybridization upon secondary contact. In *An. gambiae*, because migration included a 21 Mb inversion on chromosome arm 2L (7, 13) that is a known target of spatially varying selection (44), it is likely that at least some of the introgression was adaptive. In the *An. gambiae* complex introgression of inversions and other variation is thought to have facilitated expansion of the species range, allowed more efficient exploitation of different niches, and led to increased population density and longevity—characteristics of vectorial capacity (41). Evidence for adaptive introgression is lacking in *An. funestus* thus far, but our data suggest that the expansion of this species into its current geographic range across most of tropical Africa was subsequent to receiving 31.6 Mb of introgressed variation from *An. parensis* ∼13 Kya. For *An. funestus,* and maybe malaria vectors or disease vectors more broadly (45), this injection of genetic diversity—which plausibly has greater phenotypic consequences when the donor is a nonsister species—may have facilitated adaptation to new and anthropogenically modified environments, leading to geographic range expansion and enhanced vectorial capacity.

In animals with heteromorphic sex chromosomes, the X chromosome tends to be more resistant to introgression than the autosomes, owing in part to higher densities of incompatibility and local adaptation loci on the X (or Z) chromosome (46–50). Historical introgression in the *An. gambiae* complex conforms to this pattern as massive gene exchange between one species pair (the ancestor of the *An. gambiae* clade and *An. arabiensis*) was mainly autosomal (7). The distal X chromosome—a region distinguished by five overlapping fixed inversion differences between the *An. gambiae* clade and *An. arabiensis*—was protected from gene exchange, presumably due to both suppressed recombination conferred by the inversions and selection against introgression of incompatibility loci. These results are consistent with laboratory crossing experiments showing that certain autosomal chromosomal inversions can be introgressed between species and subsequently maintained as stable heterotic polymorphisms, while heterospecific X chromosome inversions are rapidly eliminated (51). In light of this general trend and our previous findings in the *An. gambiae* complex, we were surprised to find no strong topological discordance nor striking quantitative differences in introgression between the X chromosome and the autosomes in the AFC. One factor that may help account for this difference is that in all AFC species studied here whose karyotypes have been characterized, the X chromosomes are homosequential (16, 23, 24, 52), which should allow for greater recombination on the X chromosome relative to chromosomes that differ by fixed inversions. The absence of fixed inversion differences on the X chromosome between AFC species is suggestive. In the *An. gambiae* complex, extensively sympatric species differ by fixed inversions on the X chromosome, while species with nonoverlapping distributions and possible vicariant origins, harbor homosequential X chromosomes (53).

Speculation about the role of geography in species divergence is difficult even in the *An. gambiae* complex, but much more so in the AFC where almost all foundational knowledge about historical biogeography, current species distributions, and bionomics is absent or scant and outdated. With this important caveat, we present a working hypothesis consistent with historical climatic patterns in Africa at the time of the AFC radiation and the known biology of AFC species. During the Middle and Late Pleistocene, the climate of Africa featured repeated oscillations in temperature and rainfall linked to glacial–interglacial cycles (54). The climatic shifts between humid-warm phases (pluvials) and arid-cool phases (interpluvials) were especially intense between 115 and 90 Kya, resulting in megadroughts (55, 56) that repeatedly expanded and contracted Africa’s biomes and initiated population vicariance events (57). Taking into account the shared preference of AFC species for breeding among vegetation at the edges of lakes or slow-moving streams (21, 22, 58)—habitats already quite patchily distributed even in a mesic climate—we suppose that speciation in the AFC was allopatric and driven by arid interpluvials, but that alternating episodes of mesic pluvials could have facilitated secondary contact and hybridization and contributed to the long-term persistence of variation due to admixture (59).

In contrast to sex chromosomes, mtDNA commonly crosses species boundaries even in the absence of detectable nuclear introgression (60–62). Given this tendency, it is not surprising that we found evidence consistent with mtDNA introgression in the AFC. Although other explanations are possible and not mutually exclusive, introgression probably contributes to mtDNA paraphyly (Fig. 1*B*), and mtDNA capture most likely explains the coexistence of distant mtDNA lineages within the same species, observed both in *An. funestus* and *An. funestus-like* (shaded boxes 1 and 2, Fig. 1*B*). In the absence of alternative genomic resources, it has been common among vector biologists to employ mtDNA to make inferences about anopheline population structure, phylogeography, and even interspecific species relationships. Recently, the complete mtDNA genomes of 43 mosquitoes morphologically identified as *An. funestus* were sequenced and assembled from three localities in Southern and Central Africa (63). Bayesian phylogenetic reconstruction of these sequences revealed two deeply diverged lineages, coexisting in two of the sampling locations. The authors interpreted their findings in terms of intraspecies genetic relationships and population differentiation (63), but a reanalysis of these data together with our own reveals that the lineages described in the former study are representative of interspecific mtDNA divergences in the AFC (*SI Appendix*, Text S12 and Fig. S23). The knowledge that there has been extensive introgression between species in the *An. gambiae* complex (7) and now the AFC cautions against exclusive use of mtDNA to infer intraspecies or even interspecific relationships in closely related anopheline mosquitoes.

As African countries progress along the road toward malaria elimination, there is a growing recognition that control of the major vector species did not interrupt local transmission but instead uncovered persistent “residual” malaria transmitted by lesser-known outdoor biting species (64, 65). By itself, this situation emphasizes the importance of expanding the research emphasis to lesser vectors. Our study provides further impetus for broadening the focus, showing that the evolutionary history of a major vector species in an understudied species complex has been strongly impacted by introgression from minor and nonvector species with major consequences for malaria transmission. The time is now ripe to pivot from asking “What makes the world’s primary malaria vectors so good?” (*sensu*, ref. 66) to asking “What makes the difference between a good and a bad vector?” from a genomic and evolutionary perspective (67). High-quality reference genomes for all members of malaria vector species complexes, not only the primary vectors, is a tractable first step in that direction. Here we leveraged the recently upgraded *An. funestus* genome assembly AfunF3 (28) to generate de novo reference assemblies from species inside and outside the group that lacked these genomic resources and performed additional genome sequencing. Beyond our immediate results, these resources will support much needed future studies of the AFC. New advances in sequencing technologies (e.g., ref. 68) will lead to improved assemblies that may allow further insights into the distribution of introgression blocks along the genome and the identification of adaptive introgression. Whether introgression has played a wider role in the origin of other dominant malaria vectors beyond *An. gambiae* and *An. funestus* remains to be investigated. Our current hypothesis concerning the species branching order and reticulations in the AFC satisfactorily explains conflicts in previous mitochondrial phylogenies and provides a testable framework to underpin a deeper understanding of the origin of vectorial capacity in the AFC.

## Materials and Methods

Please see the *SI Appendix* for detailed information about: 1) sample information; 2) de novo genome assembly; 3) mitochondrial genome assembly; 4) whole genome alignments; 5) population genomics and variant calling; 6) phylogenetic reconstruction; 7) species networks using *D*-statistics and admixture graphs; 8) model selection of introgression hypotheses using random forests; 9) estimating introgression and divergence timing using ABC; 10) identifying genomic regions of introgression by machine learning; 11) detecting introgression using branch lengths; 12) introgression and inference from mtDNA.

## Supplementary Material

Supplementary File

## Data Availability

DNA sequence data have been deposited in GenBank, https://www.ncbi.nlm.nih.gov/genbank. Genome assemblies and sequence reads have been submitted to the National Center for Biotechnology Information, NIH, https://www.ncbi.nlm.nih.gov/ (accession nos. PRJNA646526 and PRJNA531511). The mitochondrial genome alignments have been submitted as a pop set for each species (*An. funestus* [MT917167–MT917182], *An. funestus-like* [MT917158–MT917162], *An. vaneedeni* [MT917128–MT917137], *An. longipalpis C* [MT917148–MT917157], *An. parensis* [MT917138–MT917147], *An. rivulorum* [MT917163–MT917164], *An. species A* [MT917165–MT917166]). VCF files of field-collected individual samples (DOI: 10.6084/m9.figshare.13017518), phylogenetic trees (DOI: 10.6084/m9.figshare.13017488), and feature vectors (DOI: 10.6084/m9.figshare.13017506) are available on https://figshare.com/. The complete models (with all of the demographic data), distributions of both rho and pi (in text and graphic formats), the observed statistics and all custom python scripts, including those used to run the ABC and ABCrf analyses conducted in this study are available at GitHub, https://github.com/stsmall/abc_scripts/.
