# Supplementary material for: Radiation with reticulation marks the origin of a major malaria vector
Source: Proc Natl Acad Sci U S A. 2020 Dec 1;117(50):31583–90. doi: 10.1073/pnas.2018142117 (PMC7749319; doi:10.1073/pnas.2018142117)
Supplement: Supplementary File [file pnas.2018142117.sapp.pdf]

PNAS
www.pnas.org

Supplementary Information for

Radiation with reticulation marks the origin of a major malaria vector.

Scott T. Small[1*], Frédéric Labbé[1], Neil F. Lobo[1], Lizette L. Koekemoer[3], Chadwick H. Sikaala[4], Daniel E. Neafsey[2], Matthew W. Hahn[5], Michael C. Fontaine[6,7], Nora J. Besansky[1*].

* Correspondence to: ssmall2@nd.edu (S.T.S.); nbesansk@nd.edu (N.J.B.).

This PDF file includes:

Supplementary text S1 to S12
Figs. S1 to S23
Tables S1 to S14
SI References

Supplementary Information Text

S1. Sample Information (Fig. S1, Table S1)

S2. *De novo* genome assembly (Fig. S2, Table S2)

S3. Mitochondrial genome assembly

S4. Whole Genome Alignments (Fig. S3, Table S3)

S5. Population genomics and Variant Calling (Figs. S4-S11, Tables S4-S5)

S5.1. Variant calling and filtering

S5.2. Interspecific demographic inferences for models and simulations

S5.3 Nucleotide diversity, Tajima's *D*, and site frequency spectrum

S5.4. Genome-wide recombination rate

S5.5. Population variation among species using PCA

S5.6. Population structure and pairwise genetic distance in *An. funestus*

S5.7. Divergence times among *An. funestus* populations

S6. Phylogenetic reconstruction (Figs. S12-S14)

S6.1. Mitochondrial genome phylogeny

S6.2. Neighbor-joining tree using SNPs from the nuclear genome

S6.3. Window-based genome phylogenies

S7. Species networks using *D* statistics and admixture graphs (Figs. S15-S16, Table S6)

S8. Model selection of introgression hypotheses using random forests (Fig S17, Tables S7-S10)

S9. Estimating introgression and divergence timing using ABC (Table S11)

S10. Identifying genomic regions of introgression by machine learning (Figs. S18-S22, Tables S11-S13)

S11. Detecting introgression using branch lengths (Table S14)

S12. Introgression and inference from mtDNA (Fig. S23)

SI References

S1. Sample Information (Fig. S1, Table S1)

Specimens were obtained from five of seven recognized *An. funestus* complex species (AFC) (1), an undescribed species (*An. species A*) morphologically similar to *An. funestus* as previously reported (2-4), and the outgroup *An. rivulorum* (Table S1). DNA was extracted from individual mosquitoes using a CTAB protocol (5) and then molecularly identified to species level using rDNA-based PCR assays (6, 7). Automated library preparation and sequencing took place at the McGill University and Génome Québec Innovation Centre (Montreal, Canada) as described in *SI Appendix,* Text S2 (for *de novo* genome assembly) and *SI Appendix,* Text S5 (for re-sequencing). Molecular species identifications were later confirmed through read mapping to ribosomal ITS2 reference sequences (Fig. S1).

S2. *De novo* genome assembly (Fig. S2, Table S2)

DNA was quantified via fluorometry (PicoGreen) and two representative female mosquitoes from each species with > 500 ng total genomic DNA were selected for individual library preparation. Libraries were prepared from inserts selected at 400 bp to ensure a 50 bp overlap with 250 cycle paired-end sequencing, performed on eight lanes of the Illumina HiSeq 2000.

Adapter sequences and low-quality bases were removed from sequencing reads using trim_galore (github.com/FelixKrueger/TrimGalore). Read pairs with one read shorter than 75 base pairs were removed. Trimmed reads were decontaminated by aligning to a custom file of bacteria (*Pantoea sp., Asaia bogorensis*, *Enterobacter asburiae*, *Klebsiella oxytoca*, *K. variicola*, and *Pseudomonas aeruginosa*) and PhiX genomes using BWA v.0.7.15 (8), with only unmapped read pairs retained. Processed reads (trimmed and decontaminated) were then aligned to the assembled con-specific mitochondrial genome (*SI Appendix,* Text S3); mapped read pairs were used for mitochondrial DNA (mtDNA) analysis while unmapped read pairs were retained for nuclear genome assembly.

Genome assembly used W2RAP (9). Optimum kmer size was determined by comparing N50 among runs using different kmer sizes with a range of 200 to 260 in increasing values of 8. The optimum kmer was then defined as the kmer that maximized N50. Contigs shorter than 1,000 bp were removed from the assembly before N50 calculation.

Assemblies were evaluated using BUSCO v.2 (10). Duplicate contigs not collapsed during assembly were identified using redundans v.0.12 (11). BUSCO was run a second time on the reduced set to verify that removal of redundant contigs did not affect assembly completeness. The reduced contig set produced by running redundans was blasted against the NCBI non-redundant nucleotide database (accessed Feb 2017). Only contigs matching *Anopheles* or Diptera were retained for scaffolding.

Contigs were aligned to an early chromosome scale version of the *An. funestus* reference genome (12) (NCBI: SAMN15857330) using progressiveCactus v.0.1 (13, 14). Resulting alignments were used in ragout v.2.0 (15) to assign scaffolds to chromosomes (Fig. S2, Table S2). Chromosome level scaffolds were evaluated using BUSCO and scaffold N50. Of the two *de novo* assemblies attempted per each species, the one with the higher BUSCO and scaffold N50 was retained and designated as the species-specific genome assembly (Table 1). The species-specific assemblies were then repeat-masked using RepeatMasker v. 4.0.7 (16) with a custom repeat file (17).

S3. Mitochondrial genome assembly

We assembled 12 mtDNA genomes [two each from five species: *An. funestus-like*, *An. longipalpis C*, *An. parensis*, *An. rivulorum*, *An. vaneedeni;* one from *An. species A*, and one from *An. funestus* representing clade 2 mtDNA (18)] from the 250 bp paired-end reads, using the *An. funestus* mtDNA genome assembly as a reference [GenBank Accession No. DQ146364; (19)] following (20). This yielded 12 mtDNA assemblies that were annotated and examined for premature stop codons using Geneious v.7 (https://www.geneious.com). One mtDNA assembly per species was submitted to GenBank along with the genome assembly (see Table 1 for accessions). Next, sequencing reads from 42 individually re-sequenced mosquitoes were aligned to their con-specific mtDNA reference genomes with BWA. Duplicates were identified with MarkDuplicates in Picard Tools

(http://broadinstitute.github.io/picard/). Variants were called using GATK v.3.5 and HaplotypeCaller. Resulting VCF files were converted to consensus sequences in FASTA format using a custom python script, vcf2fasta-mt.py. For each species an alignment of the mitochondrial genomes was uploaded to NCBI as a pop-set (see Data availability).

S4. Whole Genome Alignments (Fig. S3, Table S3)

The new *de novo* assemblies were aligned together with the *An. funestus* reference (Fig. S3, Table S3) using progressiveCactus v 0.01 (14), to provide a lift-over table (coordinate translation) into a common coordinate system based on the genome coordinates of *An. funestus*. Prior to genome alignment, masked sites were represented as lower-case (soft-masked) and missing sites were coded as 'N'. A guide tree was constructed in BEAST2 v.2.5 (21) using 100 orthologs as previously identified by the program BUSCO.

Duplicate alignment blocks were removed using HalTools v.1.2 (22) and converted to multiple alignment file (MAF) format. The MAF was projected to the *An. funestus* coordinates using maf_project v.12 in TBA (23). Then the MAF was parsed using MafFilter v.2.3 (24), first by chromosome (SelectChr), and then only retaining alignment blocks that contain all species (Subset). Alignment blocks were merged into a single alignment block with continuous coordinates by adding 'N' characters using MafFilter (Merge).

The HAL file from progressiveCactus and HalTools was then used to generate a lift-over table to allow projection of genomic locations between the individual AFC species and the *An. funestus* reference genome. A custom python script, liftover.py was used to re-orient alleles and create the projected VCF from the lift-over table (hereafter, the lift-over VCF). Phased information was then added from the con-specific VCFs. All resulting VCFs were then merged into a single VCF using BCFtools v.1.6 (25). The final merged VCF consisted of 160 Mb of accessible sites with 122.78 Mb of sites also having a genotype called for the outgroup *An. rivulorum*.

S5. Population genomics and Variant Calling (Figs. S4-S11, Tables S4-S5)

In addition to assembling new reference genomes, we also individually re-sequenced the genomes of 42 additional mosquitoes in the AFC (Table S1). For *An. longipalpis C*, *An. parensis*, and *An. vaneedeni*, an additional eight specimens were re-sequenced per species. For *An. funestus-like*, three additional individuals were re-sequenced. For *An. funestus*, 15 individuals were re-sequenced from five different countries, including six individuals carrying clade 2 mtDNA (18) (Table S1). No additional individuals were sequenced for *An. rivulorum* and *An. species A*.

All re-sequencing libraries were prepared at McGill University and Génome Québec Innovation Centre (Montreal, Canada) and sequenced on 12 lanes of the HiSeq X with 150 paired-end cycles. Sequencing reads were processed as described above (*SI Appendix,* Text S2), except that con-specific nuclear and mtDNA genome assemblies were used as a reference for mapping with BWA.

S5.1 Variant calling and filtering

Variants were called separately for each individual mosquito using GATK v.3.5 (26) and HaplotypeCaller with options: --emit-ref-confidence GVCF --heterozygosity 0.01 --indel-heterozygosity 0.001 --min-base-quality-score 17. Variant filtering was done in two steps. First, the resulting GVCFs produced by HaplotypeCaller were genotyped using GenotypeGVCFs. Variants were filtered based on the following metrics: QD < 5, QUAL < 30, DP < 14, MQ < 30, MQRankSum < -12.5, ReadPosRankSum < -8.0, FS > 60.0. Filtered GVCFs were then merged into a single species GVCF using CombineGVCFs followed by GenotypeGVCFs. Second, genotypes with a GQ < 30 and DP < 20 were marked as missing. Variant quality was evaluated using scikit-allel v1.1.0 (doi:10.5281/zenodo.2652508) following the methods described in Miles et al. (27). Sites were masked if they were identified by RepeatMasker, had read coverage outside of the bounds defined by +/- 3*sqrt(avgCov per chromosome), or identified as paralogs using the methods outlined in SNPable (http://lh3lh3.users.sourceforge.net/snpable.shtml). After masking, there were on average 160 Mb of accessible sites remaining. Data on masked and missing sites were retained to later apply masks to individual FASTA sequences. After filtering, total numbers of SNPs per species, as called against the conspecific reference, are listed in Table S4.

Filtered con-specific VCF files were statistically phased using the read-informative phasing option in SHAPEIT2 v.2.r837 (28). Phase informative read (PIR) files were generated from BAM files using the tool extractPIRs v.1.r68 available with SHAPEIT2. Switch errors were identified with the input-graph option and 100 replicates, using a custom python script. If a site switched between haplotypes in >10% of the replicates, the site was coded as unphased in the VCF.

S5.2 Interspecific demographic inferences for models and simulations

For the purpose of subsequent data simulations (*SI, Appendix*, Text S8), we reconstructed the demographic history of each AFC species using MSMC2 (29) and con-specific VCF files. Input files for MSMC2 were built using scripts from www.github.com/schiffles/msmc_tools using positive masks (callable sites) generated as described in *SI Appendix,* Text S5.1. Negative masks included homozygous positions with < 10 read coverage, and positions that were repeat-masked or missing.

For each AFC species, three individuals were combined for a total of six haplotypes. We combined haplotypes only for autosomes. We determined that the results were similar if using individual chromosome arms (e.g., tested on 2R and 3R in *An. funestus*) versus all the autosomes. Thus, we combine individuals across all autosomes. MSMC2 was run using a ρ/μ of ~2.9 (determined as described in *SI Appendix,* Text S5.5), 20 iterations, and the default time pattern. To check convergence, MSMC2 runs were repeated 10 times and the results were averaged across runs.

The msmc_tools script multihetsep_bootstrap.py was run to create input files for 20 bootstraps with a chunk size of 10 Mb and 5 chunks per four simulated chromosomes. All results were interpolated to the same generation times using a custom python script, msmc2_interpolate.py. Results from the AFC species were converted to effective population size using the *Drosophila* mutation rate of 2.8×10^{-9} per site per generation (30). The 0.025 and 0.975 quantiles of the effective population sizes estimated from the bootstrap replicates were plotted using ggplot2 (Fig. S4).

S5.3 Nucleotide diversity, Tajima's D, and site frequency spectrum

Nucleotide diversity (31) and Tajima's *D* (32) were calculated in non-overlapping windows of 10 and 50 kb using the con-specific VCFs and functions available in scikit-allel. The unfolded site-frequency spectrum (SFS) was calculated using the program *est-sfs* (33) using *An. rivulorum* and *An. species A* as outgroups to polarize the allelic state. The scaled SFS was then plotted using tools in scikit-allel.

The distributions of nucleotide diversity were similar among *An. funestus* populations, but lower for other species (Fig. S5A). Similar to the results from MSMC2, *An. funestus-like* had the lowest diversity. The distribution of Tajima's *D* statistic overlapped 0 for all AFC species except *An. longipalpis C* and *An. vaneedeni,* where it was strongly negative (Fig. S5B). Consistent with this result, the site-frequency spectrum in *An. vaneedeni* and *An. longipalpis C* samples had an excess of low-frequency and high-frequency alleles (Fig. S5C). We provide additional plots of these values along the chromosomes in Figs. S6-S8.

S5.4 Genome-wide recombination rate

A recombination map was constructed for each autosome arm (Fig. S9) using LDJump v.0.2.2 (34) for each species with at least ten individuals (the minimum number required for phasing with SHAPEIT2 v.2.r837). For *An. funestus*, only the Mozambique population was used, as sample sizes for other populations were three individuals or fewer. FASTA formatted files were created from each con-specific VCF using a custom python script. LDJump was then run using a population look-up table constructed with LDpop (35) and based on the demographic history in *SI Appendix,* Text S5.2.

S5.5 Population variation among species using PCA

We examined the genetic structure among AFC species using principal component analyses (PCA) on 50,000 randomly chosen segregating sites (Fig. S10) using functions available in scikit-allel and the lift-over VCF. SNPs were chosen if they had no missing data, were segregating in each population, and had a minor allele frequency >10%. Principal component 1 (PC1) and PC2 accounted for on average 22% and 11.3% of the variation, respectively. On the X chromosome five distinct clusters are visible splitting individuals into predefined species. On chromosomes 2R and 3R, there was no separation along PC2 between individuals belonging to *An. longipalpis C* and *An. parensis*. This was in contrast to 3L and 2L where PC2 separated *An. longipalpis C* and *An. parensis*, but PC1 did not separate individuals of *An. funestus* and *An. funestus-like*.

S5.6 Population structure and pairwise genetic distance in An. funestus

Because we had population samples of *An. funestus* from multiple geographic locations*,* we evaluated its population structure to gain insight about its possible effect on phylogenetic and other analyses. We included *An. funestus-like* as a reference to evaluate its separation from each *An. funestus* population (Fig. S11). For each chromosome arm, genetic structure was examined among *An. funestus* populations using a PCA. PCA was constructed using 50,000 random SNPs from the lift-over VCF in scikit-allel. SNPs were chosen if they had no missing data, were segregating in each population, and had a minor allele frequency >10%.

S5.7 Divergence times among An. funestus *populations*

Population divergence times among *An. funestus* populations were estimated to infer the timing of dispersal across sub-Saharan Africa. Population divergence times were calculated using two methods. The first, using the cross-coalescent rates in MSMC2, accounts for changing effective population sizes. The median cross-coalescent time was assumed to be the divergence time between populations even if the final cross-coalescent rate did not reach a value of 0. The second approach assumes no migration between populations following divergence, and is robust to small sample sizes (36). Calculations were performed using the custom python script, divTime.py.

The average divergence time among *An. funestus* populations was ~1.18 thousand years ago (Kya) with a confidence interval of [0.29-7.00 Kya] using the median cross-coalescent time, and ~0.945 Kya [0.019-2.2 Kya] using the algorithm in (36) (Table S5). The median estimates were not significantly different (*Wilcoxon rank-sum test*, *p*-value = 0.47). Estimates among pairs that included Kenya or Zambia populations, for which there was only a single individual sampled, were highly variable from both methods.

S6. Phylogenetic reconstruction (Figs. S12-S14)

S6.1 Mitochondrial genome phylogeny

Individual consensus mtDNA genomes (*SI Appendix,* Text S3) were aligned using MAFFT v.7.394 (37). Coding sequences were concatenated after removing the AT-control region, tRNA and rRNA. A Bayesian phylogeny was reconstructed from the concatenated coding sequences using BEAST2 (21) under the GTRGAMMA model and allowing for estimation of different rates per gene but assuming the same tree. BEAST2 was run with three different starting chains each with length 100 million and combined within LogCombiner (Fig. 1B, Fig. S12).

S6.2 Neighbor-joining tree using SNPs from the nuclear genome

The NJ tree (Fig. 1C) was reconstructed using the R packages *adegenet v.2.1.1* (38, 39), *ape v.5.1* (40), *poppr v.2.7.1* (41), and *vcfR v.1.7.0* (42) based on the lift-over VCF. *An. rivulorum* was used to root the trees and node support was evaluated using 1,000 bootstrap replicates.

S6.3 Window-based genome phylogenies

Phylogenies were reconstructed along the genome using maximum likelihood in PhyML v.3.1 (43). PhyML was run on the lift-over VCF using scripts available at (www.github.com/simonhmartin/genomics_general)using the phased option to split each individual genotype into two haplotypes. Windows were selected to be 5 kb in length with at least 50 informative positions and less than 50% missing sites. This resulted in 24,556 windows of 122.78 Mb of aligned base pairs (2L: 28.115 Mb, 2R: 35.45 Mb, 3R: 27.82 Mb, 3L: 18.855 Mb, X: 12.54 Mb). Resulting trees (referred to as "gene trees" regardless of their protein-coding content) were then pruned at their tips, to include only one individual per species selected at random using the custom python script, pruneTips.py. This resulted in 24,556 phylogenetic trees with one tip to represent each species. The proportion of each tree on each chromosome arm is shown in Fig. 2 and Fig. S13. The frequencies of commonly observed topologies on the autosomes and the X chromosome were comparable (Fig. S14).

S7. Species networks using *D* statistics and admixture graphs (Figs. S15-S16, table S6)

We built admixture graphs (44) for all the major trees illustrated in Fig. 2A and Fig. S13. First, a table of *D* statistics (45, 46) was calculated with ABBABABAwindows.py(www.github.com/simonhmartin/genomics_general)for all species triplets, using *An. rivulorum* as an outgroup (Table S6). Next, we fitted up to three admixture events using the *add_an_admixture* function in admixturegraph (44) (Figs. S15 and S16). After each admixture node was added, we sorted the resulting graphs in ascending order by the cost function. The cost function can be interpreted as the log likelihood of the edge lengths and admixture proportions as graph parameters (44). The top 10 graphs were examined to ensure that each was correctly rooted with *An. rivulorum*. Only the best scoring graph, randomly selected in case of ties, was used for the next step. We continued to add admixture events until the cost function no longer decreased. Given that we have six species, the cost function was not improved after the addition of a third admixture node, hence we stopped at three admixture events. We compared among the resulting graphs using the likelihood ratio test demonstrated at www.cran.r-project.org/web/packages/admixturegraph/vignettes/admixturegraph.html. Among all pairwise comparisons only three graphs (Fig. 3) could not be significantly rejected by the likelihood ratio test at a corrected *p*-value of 0.001.

S8. Model selection of introgression hypotheses using random forests (Fig. S17, Tables S7-S10)

The results from admixture graph were equivalent for the three network hypotheses involving introgression events projected onto tree *i*, *iii*, and *vii* (Fig. 3). To choose among the three trees, we used a model selection approach in *abcrf* v.1.8.1 (47). The program *abcrf* uses a supervised classification analysis of the competing evolutionary models based on a feature vector of population genetic summary statistics. We used a random forest approach over a strict ABC approach, to mitigate what is called the "curse of dimensionality" in the computational analysis of highly multivariate data (48).

To train the random forest classifier under our three models, we used a custom python script, abc_sims.py, which utilizes the coalescent simulation program *msmove* (github.com/geneva/msmove) and models introgression as a single pulse event. We modeled population size changes using the results from MSMC2 (*SI Appendix,* Text S5.2, Fig. S4), and represented the variance in observed mutation and recombination rates by drawing these parameter values from the empirical results obtained in *SI Appendix,* Text S5.3 and S5.4. We defined priors on divergence times using the equation $d_{xy}/(2\mu) - 2N_{anc}$ generations, where d_{xy} is the average pairwise divergence between species X and Y (Table S7) and N_{anc} is the ancestral population size (49).

We performed 100,000 coalescent simulations for each model with random parameter combinations drawn from priors defined in Table S8. Population genetic summary statistics were calculated using abc_scripts/abc_stats.py, which utilizes the two_popStatsML program of the FILET package (50) and the binned joint site frequency spectrum (51). To avoid biases caused by selection and linked selection, we calculated the observed statistics as the median value in windows of 10 kb located approximately 5 kb from defined protein coding regions on autosome arms; we did not consider the X chromosome. A comparison between non-coding windows and an approach using all windows did not yield different results.

We constructed the random forest in abcrf from 1,000 decision trees using 220 summary statistics with the lda option set to FALSE. We then used the predict function, also with 1,000 decision trees, to estimate the posterior probability of each scenario.

The confusion matrix (Table S9) demonstrates that we were able to confidently distinguish between alternative scenarios with a classification error rate of 3-5% and prior error rate of 0.0335 with 1,000 decision trees. The best model was based on tree *vii* with average of 600 votes out of 1,000 votes and a posterior probability of 0.68 (Table S10). Importance parameter values indicated that the most informative statistics were those associated with the species pairs Lik-Van, Fun-Lon, and Fun-Par (Fig. S17).

S9. Estimating introgression and divergence timing using ABC (Table S11).

We used the *abc* package (52) to estimate the divergence and introgression times under our best model, tree *vii*, with three introgression events. Priors were defined as in Table S8. The program *abc* is not able to utilize the entire vector of population genetic summary statistics we calculated for model selection (*SI Appendix,* Text S8). Therefore, we used a subsample of 20,000 simulations and estimated the contribution of each summary statistic to the parameter estimation using the regression function in *abcrf* (53). We then chose the top 25 statistics (sorted in descending order on most to least important) to inform the parameter estimates in the package *abc*.

We ran an additional 2,000,000 simulations using the custom python scripts noted in *SI Appendix,* Text S8. Posterior estimates of introgression times and divergence times were estimated in *abc* using the neural net option with tol = 0.005, sizenet = 10, and numnet = 15 (52). Cross-validation was used to examine the effect of tolerance choice on parameter estimates by running 100 cross-validation simulations and using the rejection method and a vector of tolerances, tols = 0.01, 0.005, 0.0005. Priors and posteriors were examined using the plot function in *abc*.

Results are presented in Table S11. Divergence and introgression times are presented in generations assuming a mutation rate of 2.8×10^{-9} mutations per site per generation (30). Values in parentheses are the 95% credible intervals for the value.

In addition to divergence and introgression, we estimated the current effective population size for each species using the vector of population genetic summary statistics. We allowed for each species to have a uniform prior on its current population size and defined by the 95% confidence intervals from MSMC2 (*SI Appendix,* Text S5.2). For all species the estimates were consistent with the median of the last epoch of MSMC2, except for *An. funestus* which had an effective population size twice as large as the MSMC2 estimate and *An. funestus-like* that had a population size three times larger (Table S11).

We verified that each *An. funestus* population was equally distant from *An. parensis* using pair-wise genetic distance (d_{XY}). We were particularly interested in whether *An. funestus* populations geographically closer to *An. parensis* (sampled from Mozambique, Table S1) have a smaller genetic distance from *An. parensis* potentially indicative of recent or ongoing introgression. We calculated d_{XY} in 10 kb windows along the genome using scikit-allel. We found that the pairwise nucleotide divergence was highly similar between each *An. funestus* population and *An. parensis* (d_{XY} avg 0.026, stdev 0.0004).

S10. Identifying genomic regions of introgression by machine learning (Figs. S18-S22, Tables S11-S13)

To characterize where in the genome introgression took place we used the program FILET (Finding Introgressed Loci using Extra Trees Classifiers) (50). FILET uses simulated training data in a random forest classifier to assign windows along the genome as introgressed or non-introgressed, and it can also define the direction of introgression. We chose FILET because we can train the classifier using simulations that are custom to our model for evolution of the AFC (Table S11). This allows us to consider the entire evolutionary history of the AFC with multiple introgression events and specific demographic processes.

The FILET classifier was trained on population genetic summary statistics generated using the coalescent simulator msmove (github.com/geneva/msmove) for each category (migration of species 1 into species 2, mig21; migration of species 2 into species 1, mig12; and no migration, noMig). We expanded our analyses to all pairs of species and not just the three introgression events in tree *vii* because it was possible that minor events, contributing less to the observed *D* statistics used by *admixturegraph*, may still be present among the AFC

species. Following the notation of (50), we define T_M (time since migration) as a proportion of T_D (time since divergence). We only include parameter combinations of introgression times that were as old as 75% of the divergence time for pairs of species. Introgression intensity (P_M) was defined as a pulse migration event using the flag *-ev* in msmove.

We inferred that *An. funestus* and *An. parensis* had gene flow during two different times (Events A and C, Fig. 1D). To differentiate these events, we trained the classifier on simulated data under two exclusive scenarios, one that tested migration at event A but barred it at event C, and a second under the converse. The mean divergence among *An. funestus* populations is consistent with a very recent expansion across Africa (*SI Appendix,* Text S5.7), suggesting that geographic source of *An. funestus* should not have a major impact. Accordingly, as our sample size for *An. funestus* was small for most locations (Table S1), we used the Mozambique population of *An. funestus*, for which we had six specimens.

We generated 180,000 simulations (60,000 for each migration direction and no migration) with parameter combinations drawn from uniform distributions of T_M (0, 0.75 x T_D], and P_M defined by results from the ABC analyses (Table S11). Separate simulations were performed for the X chromosome, under the assumption of an effective population size of 0.75 relative to the autosomes. We masked our simulated data to match masking in our genome data following suggestions at https://github.com/kern-lab/FILET using a custom python script, makeFILETmask.py. We withheld 10,000 simulations for construction of a confusion matrix and to verify posterior cutoffs (Table S12). In all cases the rate of false positives was very low, however some classifications (mostly involving older events) had a high rate of false negatives in at least one direction.

Population genetic summary statistics for classifying windows as introgressed were constructed for each pair of species (Fun-Lik, Fun-Van, Fun-Lon, Fun-Par, Lik-Van, Lik-Lon, Lik-Par, Van-Lon, Van-Par, Lon-Par) from phased FASTA files masked for missing data and repeat motifs. Each summary statistic was calculated in 10 kb windows with a sliding step of 1 kb, omitting any window for which > 50% of sites were masked or missing. The classifiers were trained using a feature vector of all population genetic summary statistics.

Following classification, we clustered adjacent windows showing evidence of introgression by joining consecutive windows with > 90% probability of introgression (i.e. the probability of no-introgression class <10%). Plots of genome-wide introgression are presented in Figs. S18-S22 and Fig. 4. Estimates of the amount of introgressed DNA between species pairs, and the amounts and proportions of introgression on the autosomes versus the X chromosome are presented in Table S13.

S11. Detecting introgression using branch lengths (Table S14)

As a secondary test of our introgression results we used the branch length test provided in the program QuIBL (54). Species triplets were extracted from the 24,556 phylogenetic trees representing ~122.78 Mb of aligned sequence data (*SI Appendix,* Text S6). For a given species triplet, QuIBL computes the frequency of a given triplet tree and estimates the distribution of branch lengths under the three alternate trees. We ran QuIBL on every species triplet under default parameters with number of steps (numsteps) equal to 50 and specifying *An. rivulorum* as root. QuIBL classifies triplet topologies as resulting from either ILS or introgression/speciation by fitting the branch lengths to an expected distribution under each scenario (54). We examined the triplet count for the autosomes (total trees 22,048) and X chromosome (total trees 2,508) and kept only those counts which were derived from introgression (except as noted in Table S14) by conditioning on a Bayes factor of <= -10.0 (supporting of the introgression model over ILS). To determine which arrangements were introgressed we evaluated the results against tree vii, the hypothesized species branching order (*SI Appendix,* Text S8).

S12. Introgression and inference from mtDNA (Fig. S23)

Complete mtDNA genomes from mosquitoes morphologically identified as *An. funestus* were recently sequenced and assembled from three localities in southern and Central Africa (55). The Bayesian phylogeny reconstructed from these sequences revealed two deeply diverged lineages, whose last common ancestor was estimated at ~500,000 years ago. To better understand the implication of these results in the context of our findings from the present study, we aligned the individual consensus mtDNA genomes from our study (*SI Appendix,* Text S3) together with those deposited by (55). All sequences were aligned using MAFFT (37).

Coding sequences were concatenated after removing the AT-control region, tRNA and rRNA. We reconstructed a Bayesian phylogeny from the concatenated coding sequences using BEAST2 (21) under the GTRGAMMA model, allowing for estimation of different rates per gene but assuming the same tree. BEAST2 was run with three different starting chains each with length 100 million and combined within LogCombiner. The resulting mtDNA genome phylogeny is presented in Fig. S23.

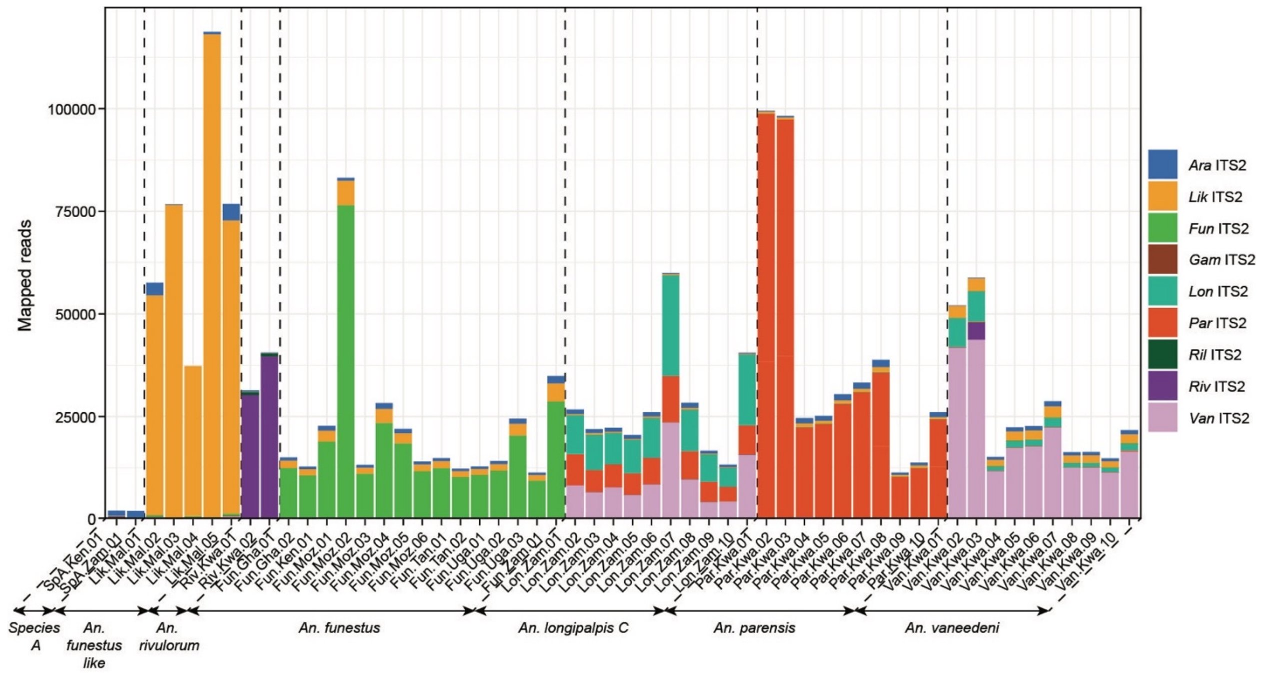

Fig. S1. ***Confirmation of species assignments through read mapping to ribosomal ITS2 reference sequences.*** *To verify species identifications, whole genome sequencing reads were mapped using BWA to a set of ribosomal ITS2 reference sequences available from Genbank: An. funestus (AF062512), An. vaneedeni (AY035718), An. funestus-like (JN994137), An. longipalpis C (EF136463, EF095767), An. parensis (AY035720), An. rivulorum (AF210724), An. rivulorum-like (JN994147), An. gambiae (KU056615), and An. arabiensis (KT160245). A reference ITS2 sequence was not available for An. species A. Shown is the number of reads that mapped to each reference ITS2 sequence for each individual mosquito. Species and specimen names are listed along the horizontal axis, with the total number of mapped reads on the vertical axis. Confirmation of species assignments is demonstrated by the high majority reads mapping to the con-specific reference. In the case of individuals identified as An. longipalpis C, ~50% of reads mapped to the reference ITS2 sequences of An. vaneedeni or An. parensis as expected, due to the previously reported ITS2 sequence similarity between An. longipalpis C and these two species (7, 56, 57). Low numbers of reads (<600) mapping to heterospecific references were recorded from all of the AFC species, and from An. species A to the An. arabiensis reference. Abbreviations: An. arabiensis (Ara), An. funestus (Fun), An. gambiae (Gam), An. funestus-like (Lik), An. longipalpis C (Lon), An. parensis (Par), An. rivulorum-like (Ril), An. rivulorum (Riv), and An. vaneedeni (Van).*

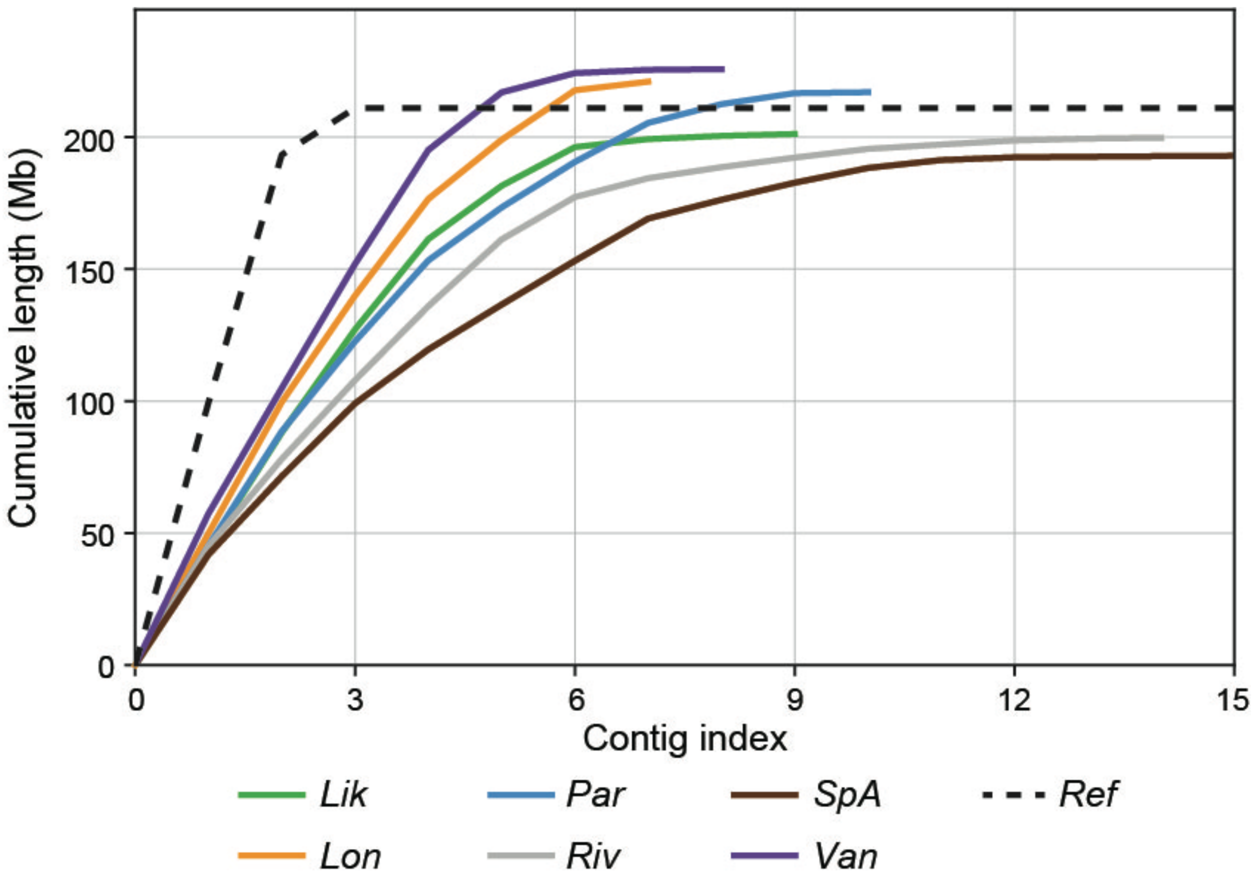

Fig. S2. ***Contig accumulation curve for de novo assemblies compared to reference AfunF3.*** *Abbreviations: An. funestus-like (Lik), An. longipalpis C (Lon), An. parensis (Par), An. rivulorum (Riv), An. species A (SpA), An. vaneedeni (Van), and reference AfunF3 (Ref).*

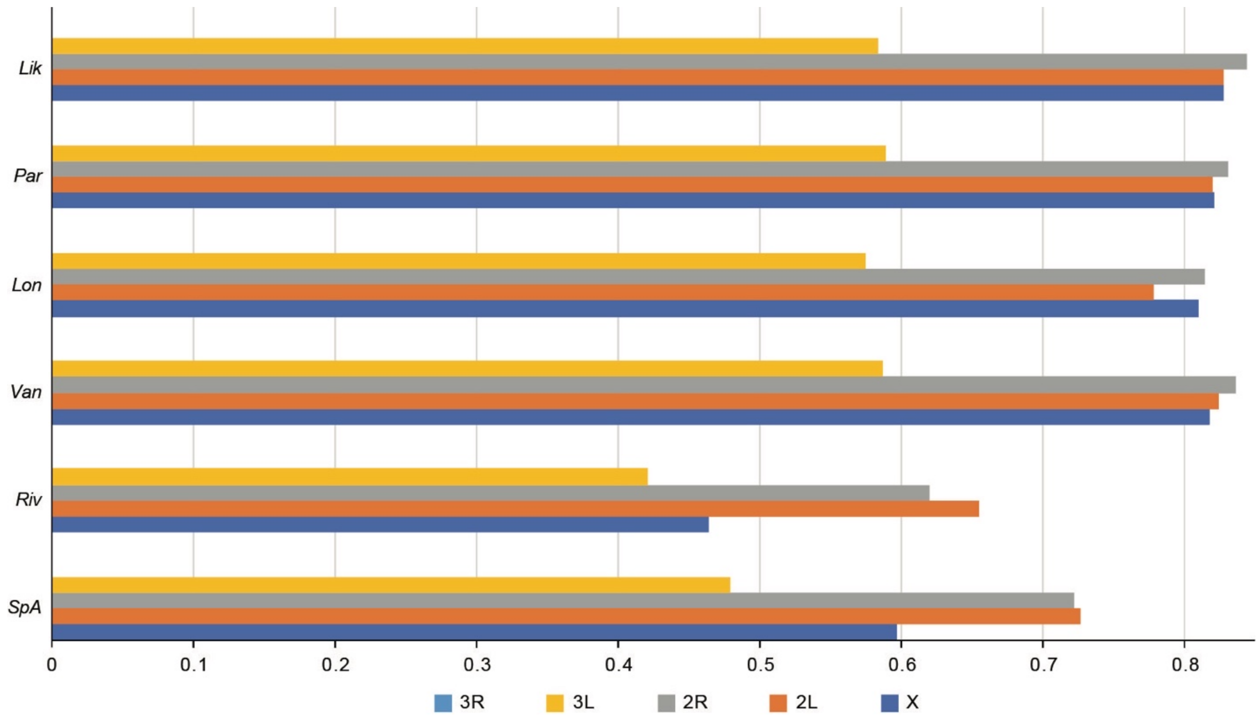

Fig. S3. ***Percent of the genome aligned from the AFC species to the reference sequence AfunF3.*** *Pairwise alignments were used for coordinate projections. Abbreviations: An. funestus-like (Lik), An. longipalpis C (Lon), An. parensis (Par), An. rivulorum (Riv), An. species A (SpA), and An. vaneedeni (Van).*

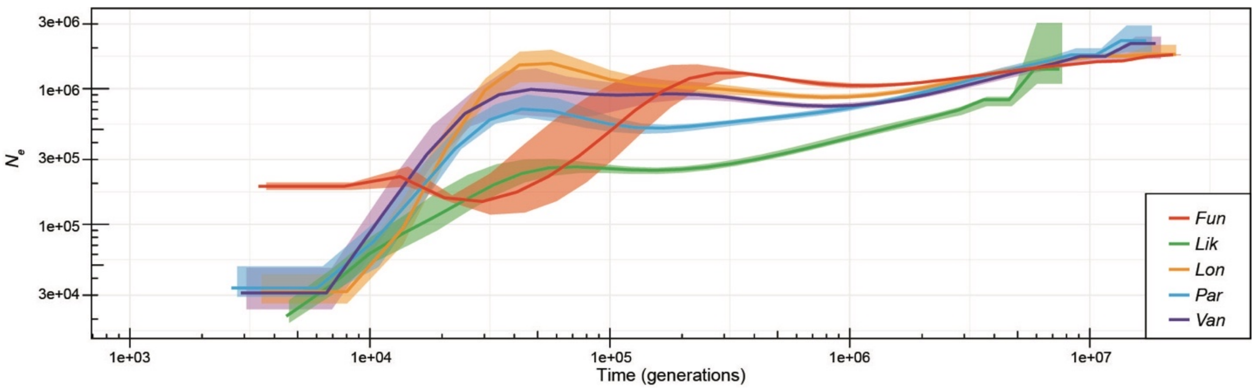

Fig. S4. ***Demographic reconstruction of AFC species using MSMC2.*** *Confidence intervals are represented as shaded regions with colors corresponding to AFC species in the legend. For An. funestus, MSMC2 was run on each population sample (Table S1) and then results were interpolated and averaged for each time epoch. The ancestral population size was estimated at 1,400,000 individuals. The most recent estimates, 3,000 generations ago, were similar for An. vaneedeni, An. longipalpis C, and An. parensis at 30,000 – 40,000 individuals. An. funestus-like had the smallest size at 20,000 individuals. An. funestus, the most widely distributed species of the AFC, had an effective population size of ~200,000 and while this was larger than the other AFC species it is smaller than current estimates for An. gambiae from some populations, e.g., An. gambiae from Uganda with a population size of ~5 million (58). Abbreviations: An. funestus (Fun), An. funestus-like (Lik), An. longipalpis C (Lon), An. parensis (Par), and An. vaneedeni (Van).*

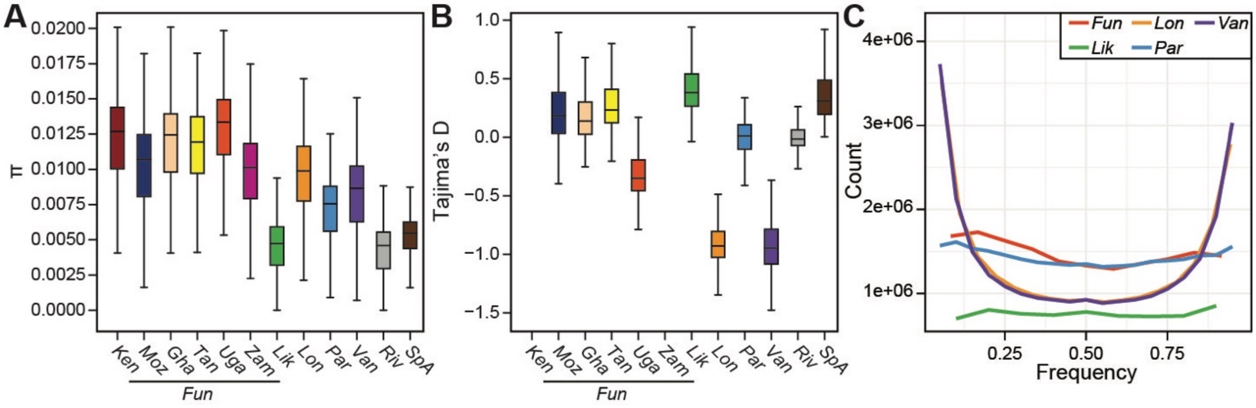

Fig. S5. ***Population genomic statistics for each AFC species.*** *(A) Box plot showing the distribution of nucleotide diversity for each species and An. funestus population. (B) Box plot showing the distribution of Tajima's D values. (C) Scaled site-frequency spectrum (SFS). The species Lon and Van (orange and purple lines) overlap in their SFS. Abbreviations: An. funestus-like (Lik), An. vaneedeni (Van), An. parensis (Par), An. longipalpis C (Lon), and An. funestus (Fun) populations from Ghana (Gha), Kenya (Ken), Mozambique (Moz), Tanzania (Tan), Uganda (Uga), and Zambia (Zam).*

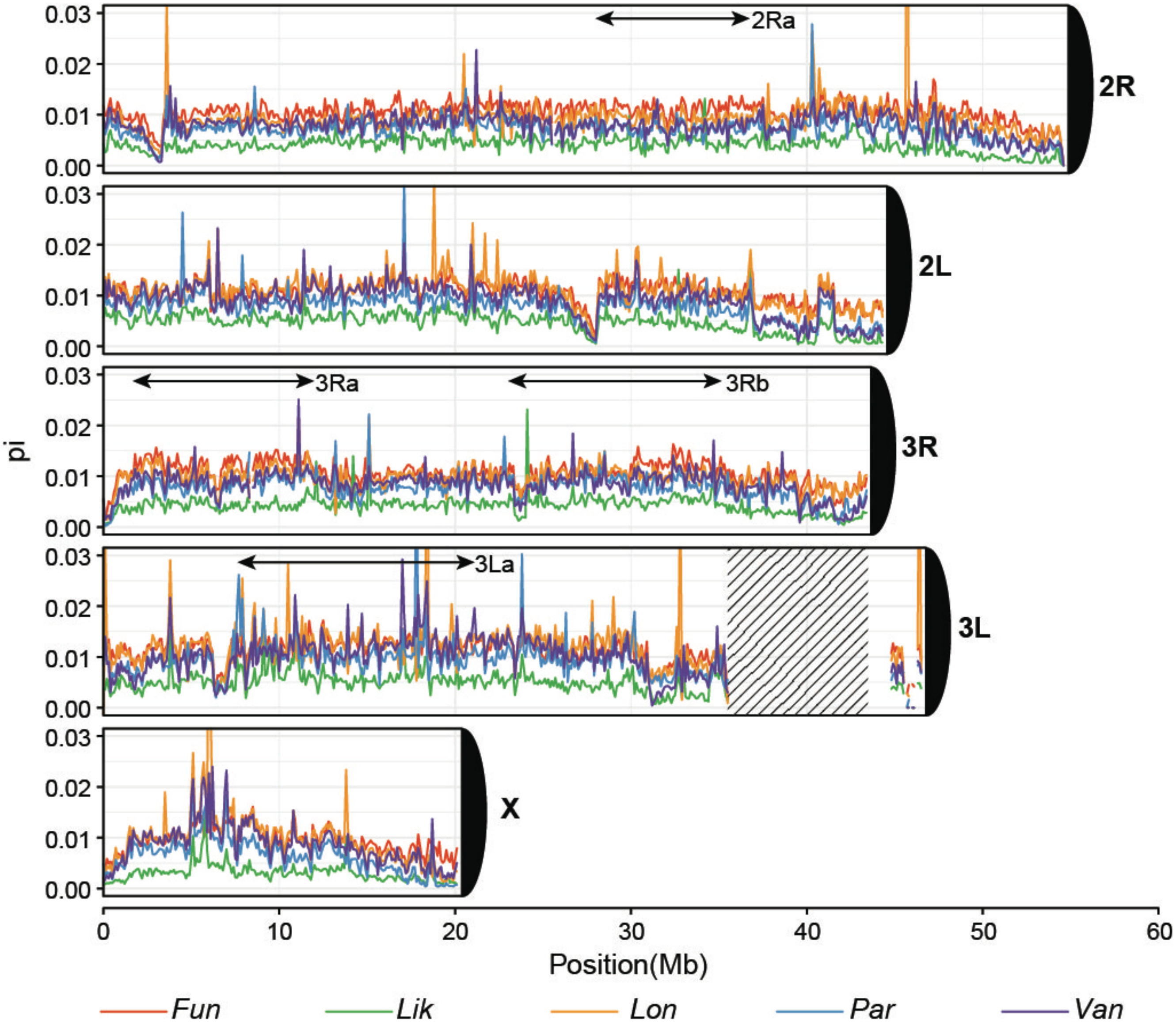

Fig. S6. ***Chromosomal distribution of nucleotide diversity in 100 kb windows for each AFC species.*** *Hatched regions represent large regions of masked sites. Each chromosome arm is oriented with the centromere on the right of the plot. Abbreviations: An. funestus (Fun), An. funestus-like (Lik), An. longipalpis C (Lon), An. parensis (Par), and An. vaneedeni (Van).*

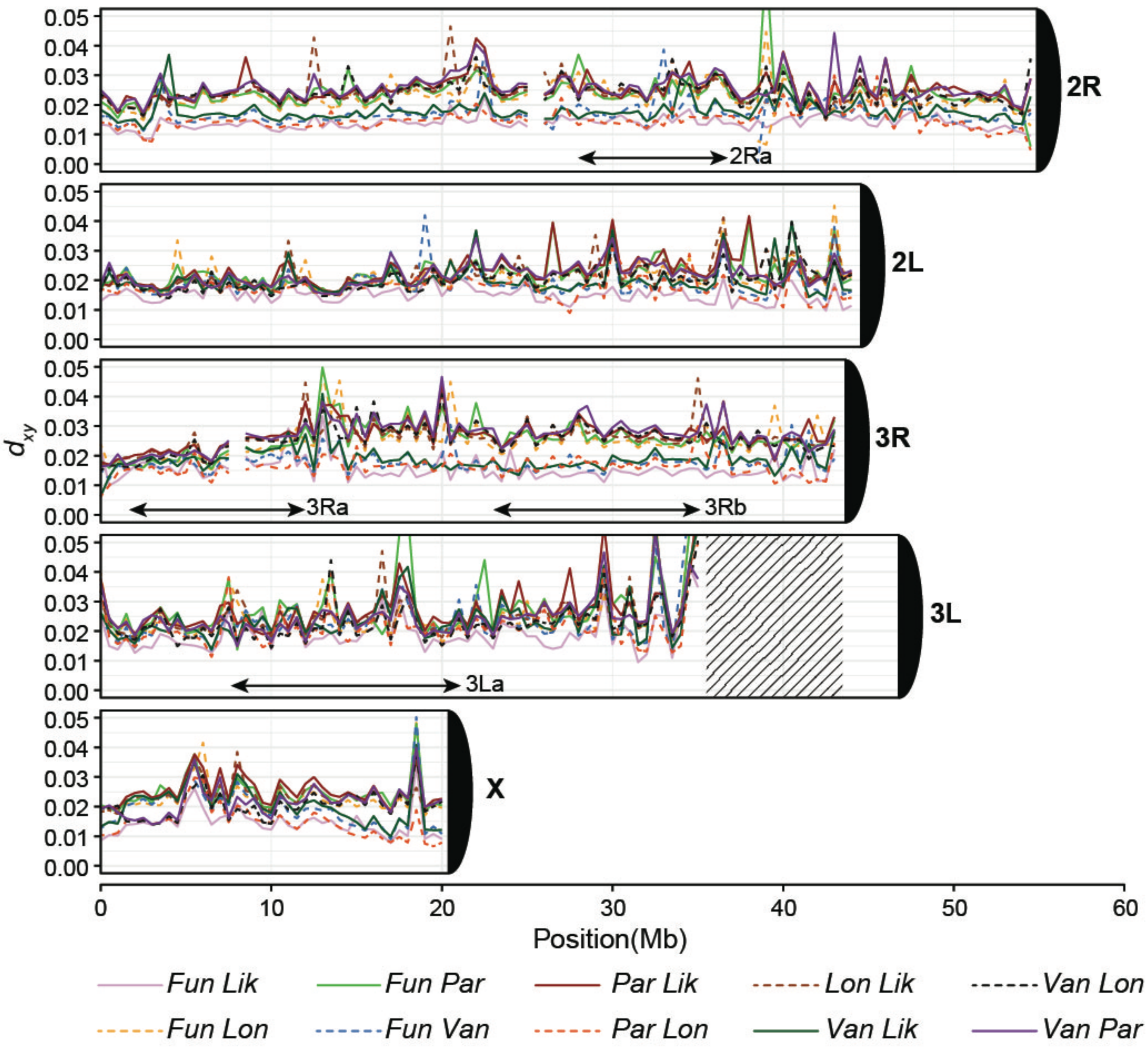

Fig. S7. ***Chromosomal distribution of pairwise differences (d_{XY}) in 100 kb sliding for each AFC species pair.*** *Hatched regions represent large regions of masked sites. Each chromosome arm is oriented with the centromere on the right of the plot. Abbreviations: An. funestus (Fun), An. funestus-like (Lik), An. longipalpis C (Lon), An. parensis (Par), and An. vaneedeni (Van).*

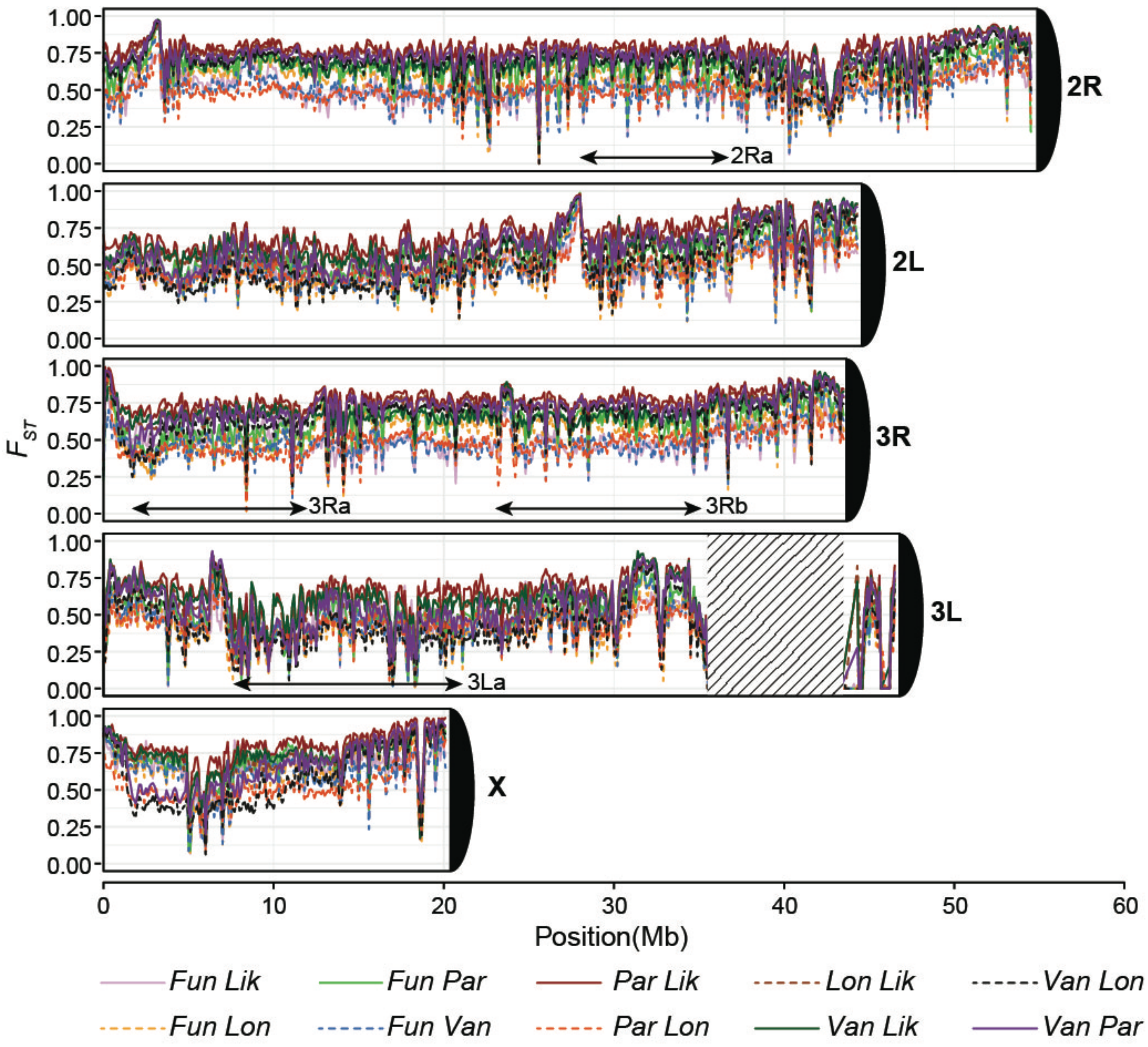

Fig. S8. ***Chromosomal distribution of the fixation index (F_{ST}) in 100 kb sliding windows for each AFC species pair.*** *Hatched regions represent large regions of masked sites. Each chromosome arm is oriented with the centromere on the right of the plot. Abbreviations: Abbreviations: An. funestus (Fun), An. funestus-like (Lik), An. longipalpis C (Lon), An. parensis (Par), and An. vaneedeni (Van).*

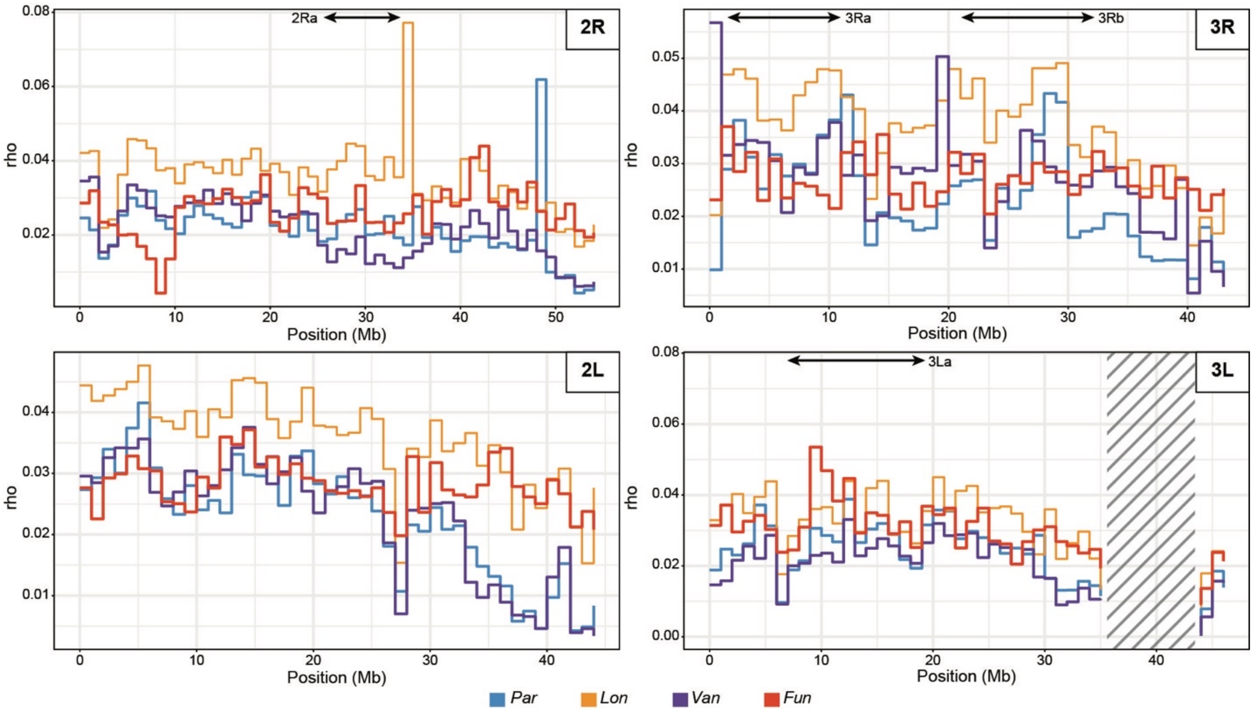

Fig. S9. ***Recombination maps for AFC species.*** *A recombination map was estimated for An. parensis (Par), An. longipalpis C (Lon), An. vaneedeni (Van), and An. funestus (Fun; Mozambique population). The mean of the population recombination parameter ρ was then estimated in 10 kb sliding windows (rolling mean). Hatched regions represent large regions of masked sites. Each chromosome arm is oriented with the centromere on the right of the plot.*

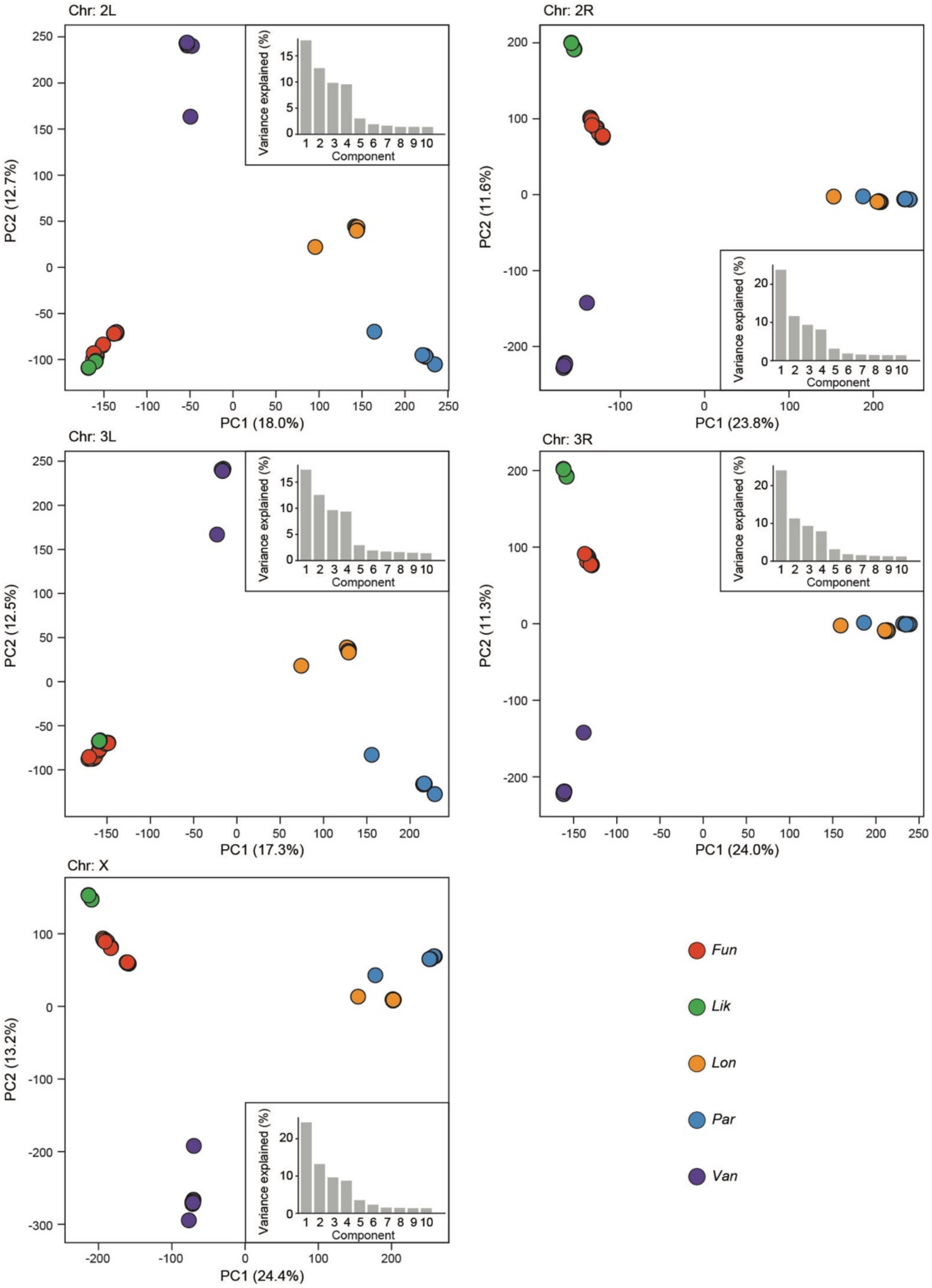

Fig. S10. *PCA plots by chromosome arm for AFC species.* *Each PCA was constructed as described in SI Appendix, Text S5.5. Colors at the bottom right of the figure correspond to species classification. Abbreviations: An. funestus (Fun), An. funestus-like (Lik), An. longipalpis C (Lon), An. parensis (Par), and An. vaneedeni (Van).*

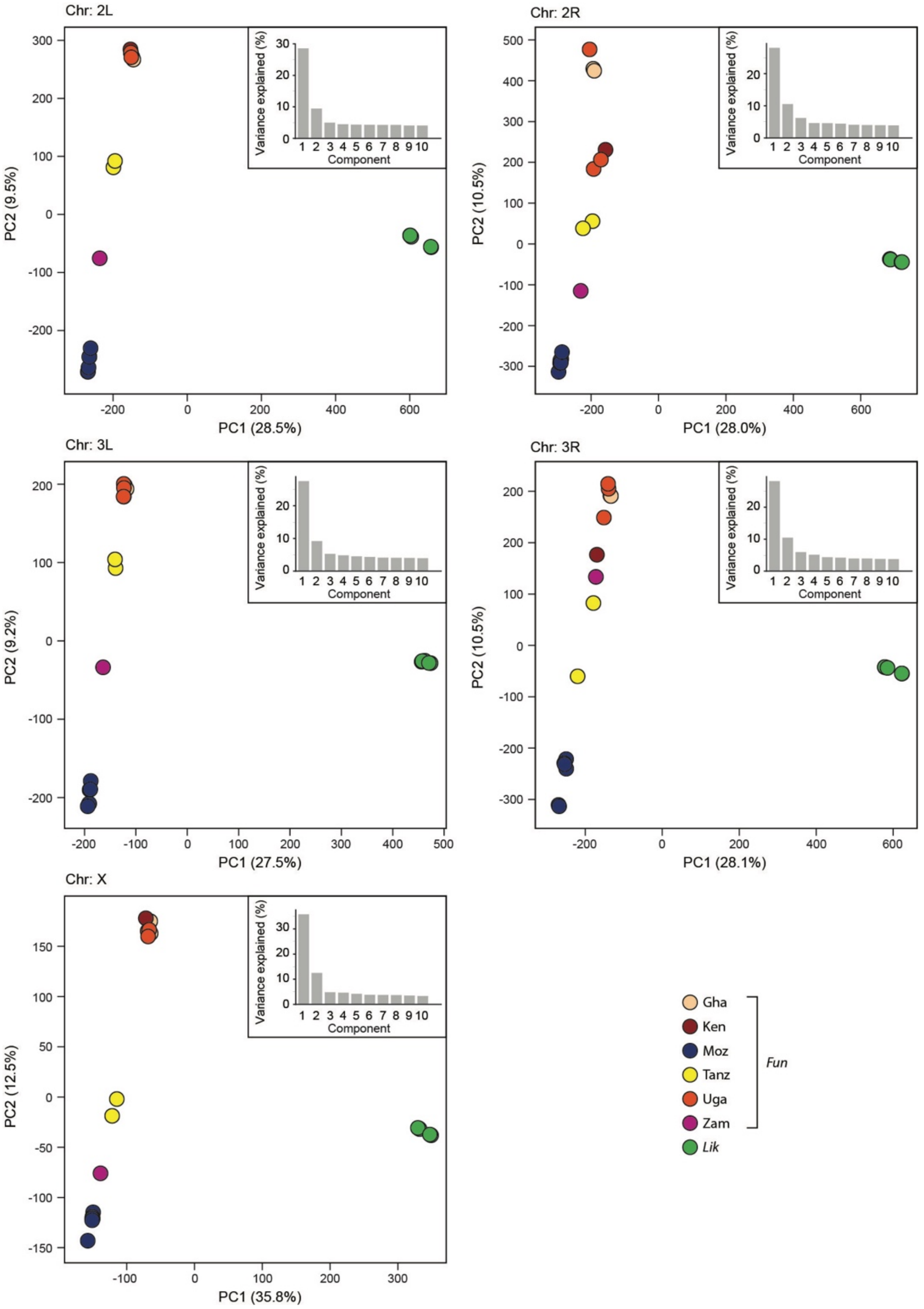

Fig. S11. ***PCA plots by chromosome arm for An. funestus populations and An. funestus-like using 50,000 segregating sites.*** *PCA supported that all An. funestus populations are equally distant from An. funestus-like. The results for each chromosome arm were consistent, where the An. funestus populations are separated from An. funestus-like along PC1 (28-35%) and the An. funestus populations along PC2 (10-12%). Abbreviations: An. funestus-like (Lik), An. funestus (Fun) population samples from Ghana (Gha), Kenya (Ken), Mozambique (Moz), Tanzania (Tan), Uganda (Uga), and Zambia (Zam).*

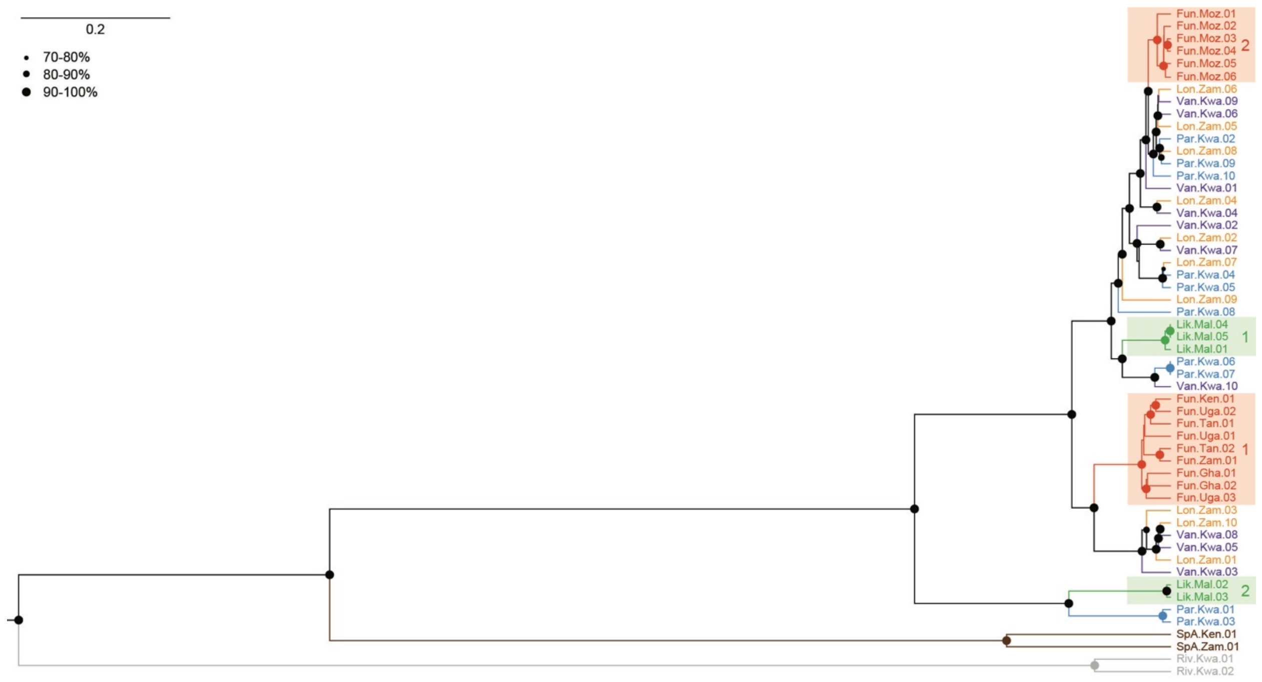

Fig. S12. ***Bayesian phylogenetic tree of the assembled mitochondrial genomes for the AFC and An. species A, with An. rivulorum as outgroup.*** *The phylogeny was reconstructed with BEAST2, using independent models for the rates of evolution in coding regions (non-coding regions were excluded). Circles at nodes correspond to posterior support values above 70%; increasing support is reflected by increasing diameter (see key in upper left). Species are color-coded. Geographic origin of individual specimens is encoded in taxon labels (see S1 Table): Kenya (Ken), South Africa (Kwa), Malawi (Mal), Mozambique (Moz), Tanzania (Tan), Uganda (Uga), and Zambia (Zam).*

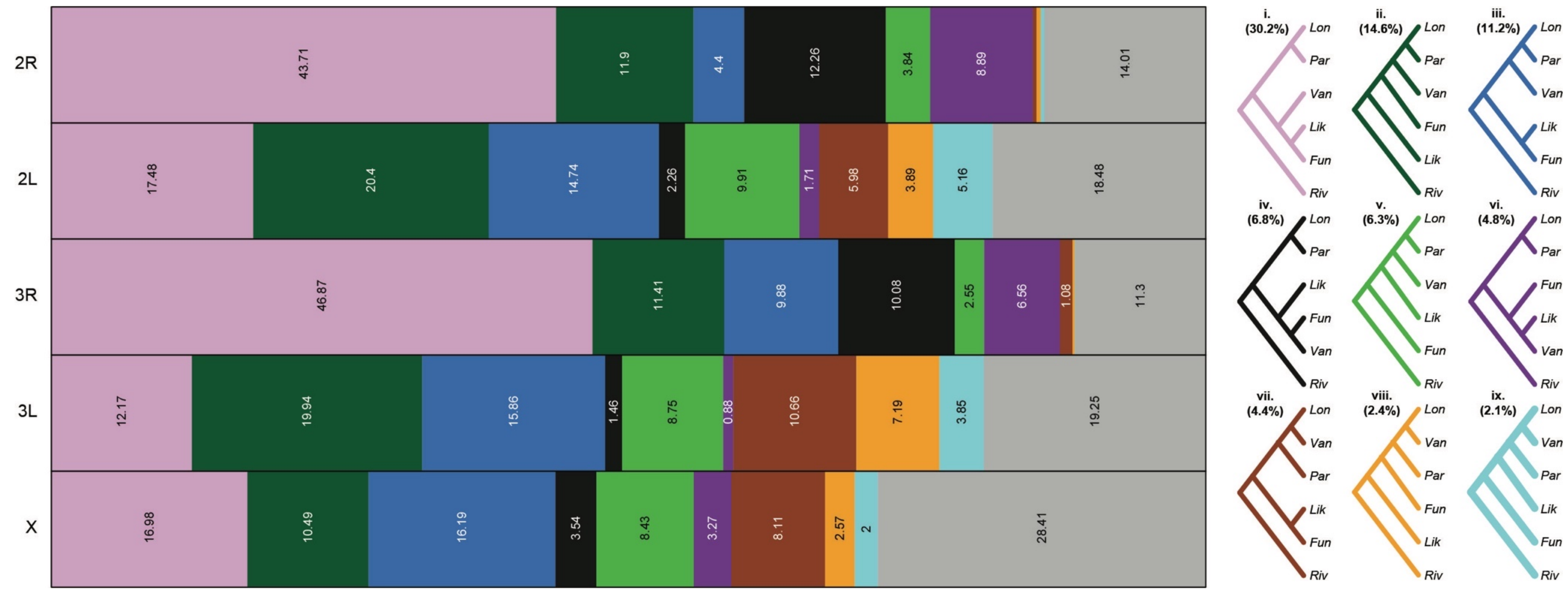

Fig. S13. ***Gene trees on each chromosome arm in the AFC.*** *Right panel shows nine topologies observed with a frequency of at least 5% on at least one chromosome arm (see Fig 2). Shown in parentheses is the total frequency of that topology across the genome (over all windows). Each topology is colored to match the stacked bar chart on the left, where blocks in each bar represent the proportion of all 10-kb windows on each chromosome arm supporting a given topology. Low frequency topologies are pooled and represented in gray. Abbreviations: An. funestus (Fun), An. funestus-like (Lik), An. longipalpis C (Lon), An. parensis (Par), An. rivulorum (Riv), and An. vaneedeni (Van).*

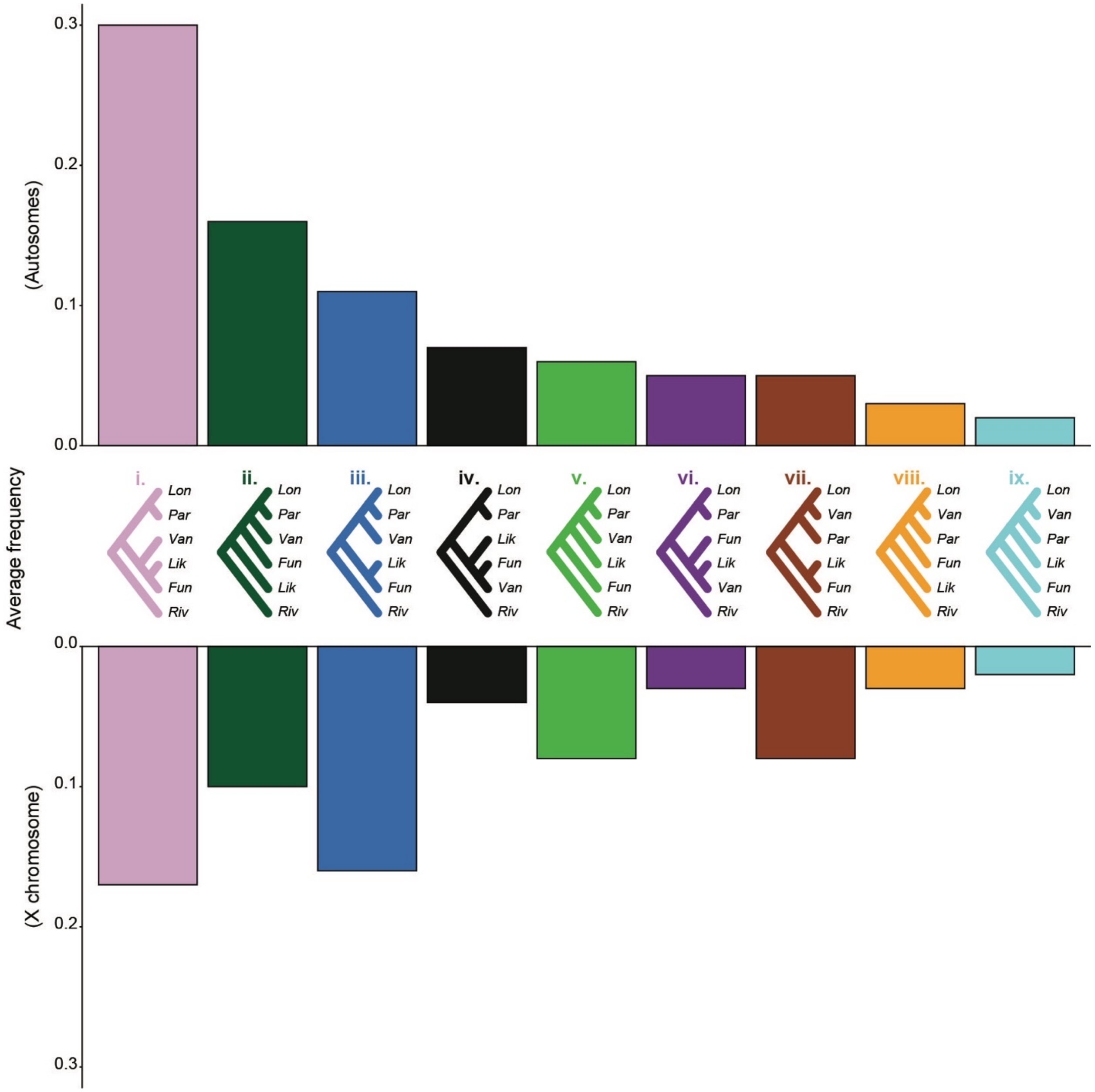

Fig. S14. ***Average frequencies of the nine major topologies on the autosomes and X chromosome.*** *The topologies are arranged in descending frequency observed on the autosomes. Abbreviations: An. funestus (Fun), An. funestus-like (Lik), An. longipalpis C (Lon), An. parensis (Par), An. rivulorum (Riv), and An. vaneedeni (Van).*

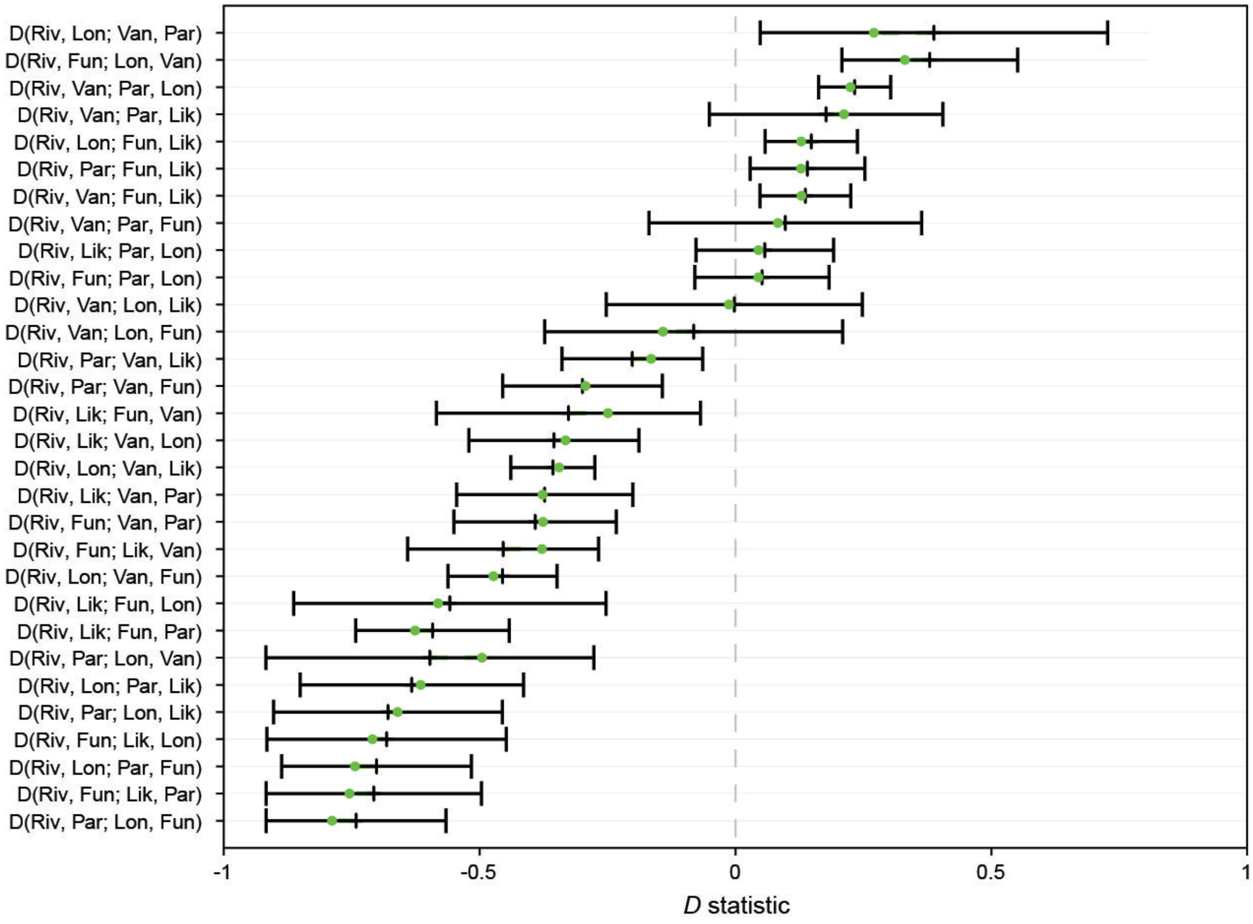

Fig. S15. ***D statistics for all species triplets using An. rivulorum as an outgroup.*** *D statistics for each triplet (X,Y,Z) with An. rivulorum (W) as outgroup (D (W,X,Y,Z)). If the D statistic is positive, the inference is that gene flow occurred between X and Y. If the Z-score is negative then the inference is that gene flow occurred between X and Z. The notch and whiskers represent the observed D value and associated Z-score for each triplet shown on the vertical axis. The green circles are the predicted values of the D statistic under tree vii with three introgression events. Abbreviations: An. funestus (Fun), An. funestus-like (Lik), An. longipalpis C (Lon), An. parensis (Par), An. rivulorum (Riv), and An. vaneedeni (Van).*

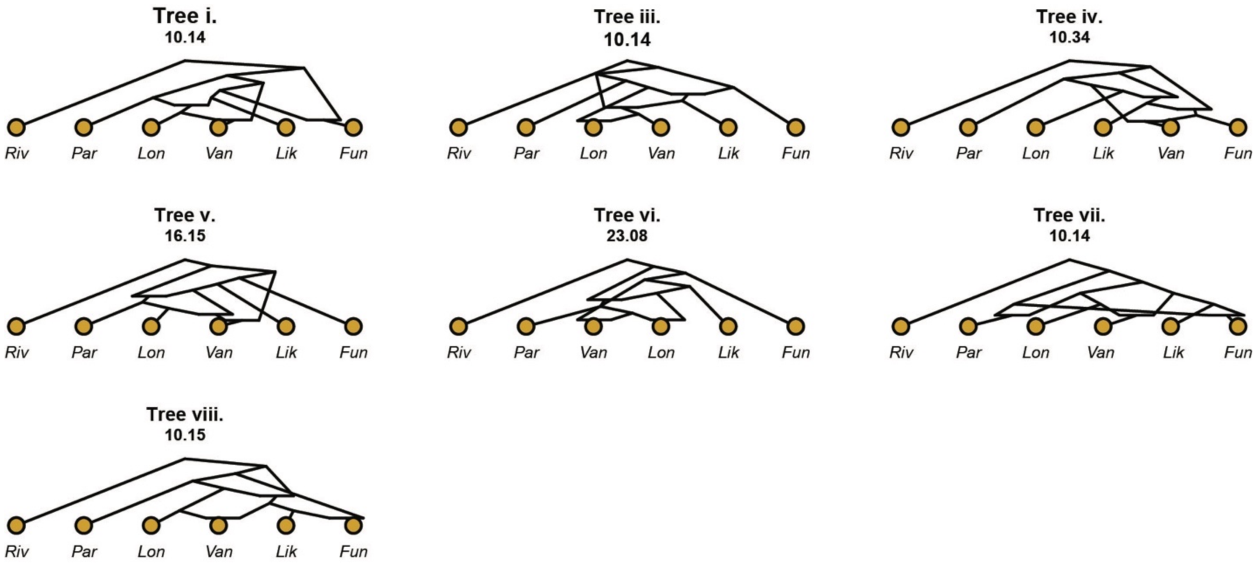

Fig. S16. ***Admixture events projected onto the major trees using admixturegraph.*** *Trees ii and ix are not shown because it was not possible to place three admixture events without moving the root node. The cost function is shown below the label. Abbreviations: An. funestus (Fun), An. funestus-like (Lik), An. longipalpis C (Lon), An. parensis (Par), An. rivulorum (Riv), and An. vaneedeni (Van).*

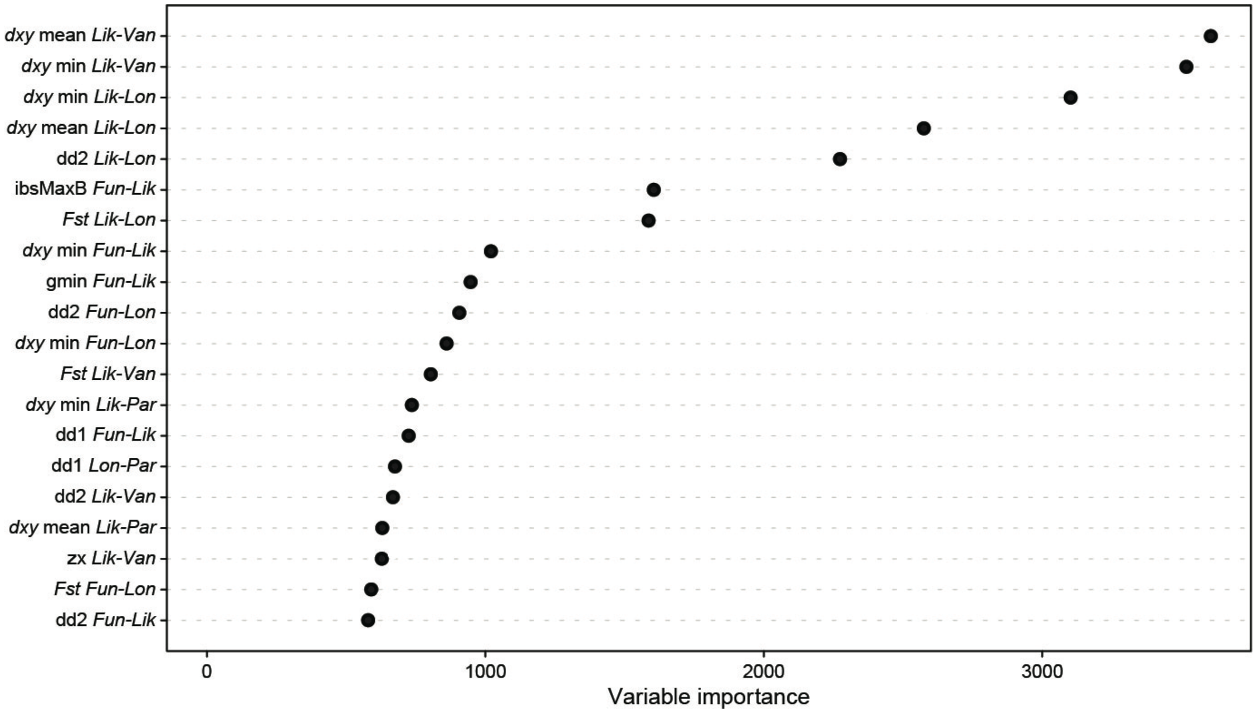

Fig. S17. ***Variable importance for model selection using abcrf.*** *Variables are given on the vertical axis and importance weights are shown on the horizontal axis. Statistic abbreviations: pairwise nucleotide difference (dxy_mean), minimum value of* d_{XY} *(dxy_min), the ratio of the minimum pairwise divergence across all cross-population comparisons [termed* d_{min} *(50)] to the nucleotide diversity in population 2, ibsMaxB (identify by state maximum length for second species), Wright's fixation index (Fst), gmin (59), the ratio of* d_{min} *to the nucleotide diversity in population 1 (dd1)(50), and average linkage disequilibrium within populations to average LD within the global population (zx)(50). Species abbreviations: An. funestus (Fun), An. funestus-like (Lik), An. longipalpis C (Lon), An. parensis (Par), and An. vaneedeni (Van).*

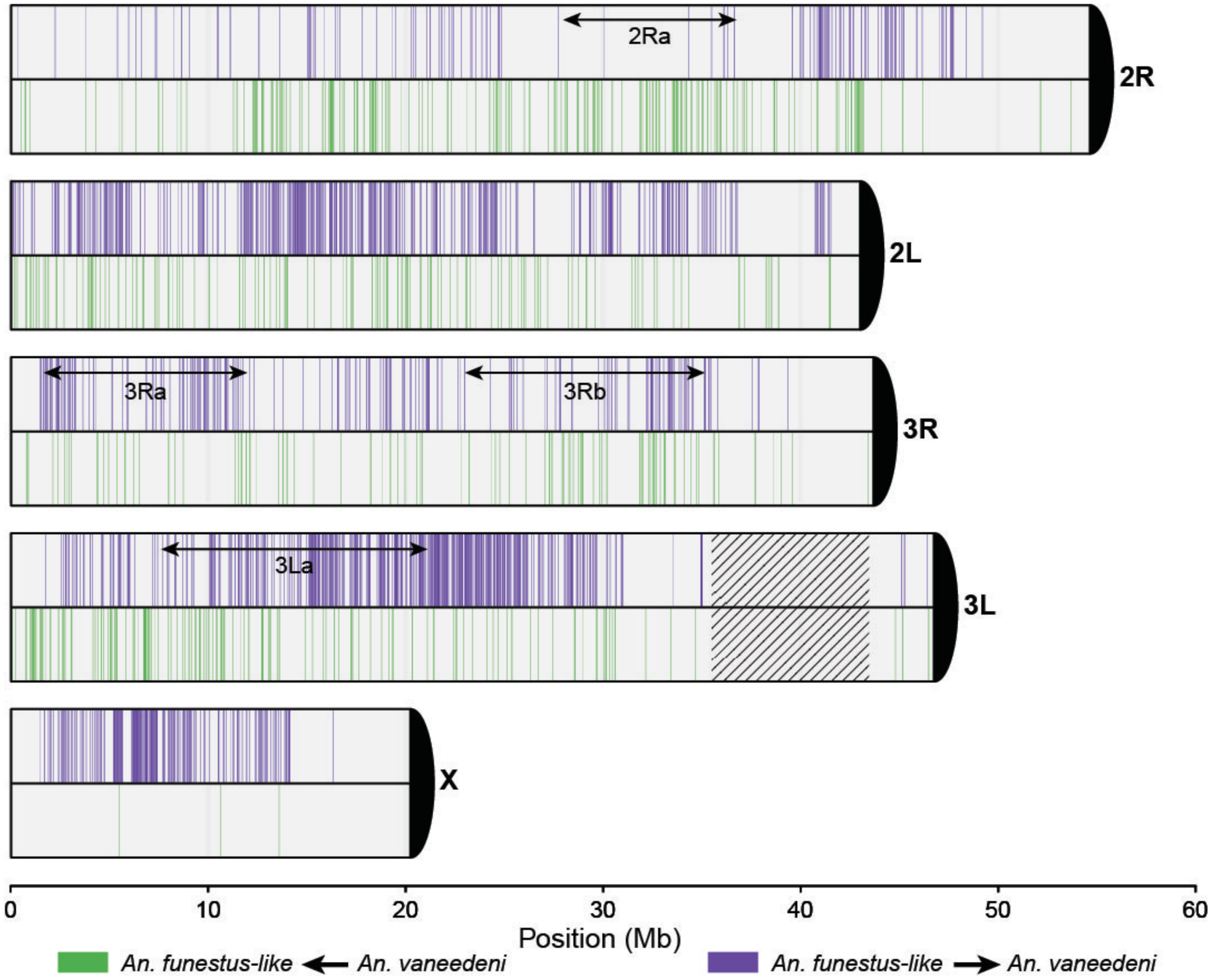

Fig. S18. ***Window-based estimates of introgression between An. funestus-like and An. vaneedeni from FILET.*** *Windows classified as introgressed at a probability of > 90% are shown on the vertical axis. Above the zero-line, purple represents regions that originated in An. funestus-like and introgressed into An. vaneedeni. Below the zero-line, green represents genomic regions that originated in An. vaneedeni and introgressed into An. funestus-like. Approximate locations of known inversions in An. funestus (3Ra, 3Rb, 3La, and 2Ra) are indicated by double-headed arrows. Centromeres are represented as black ¼ circles at the right of each chromosome arm. The hatched box represents masked region.*

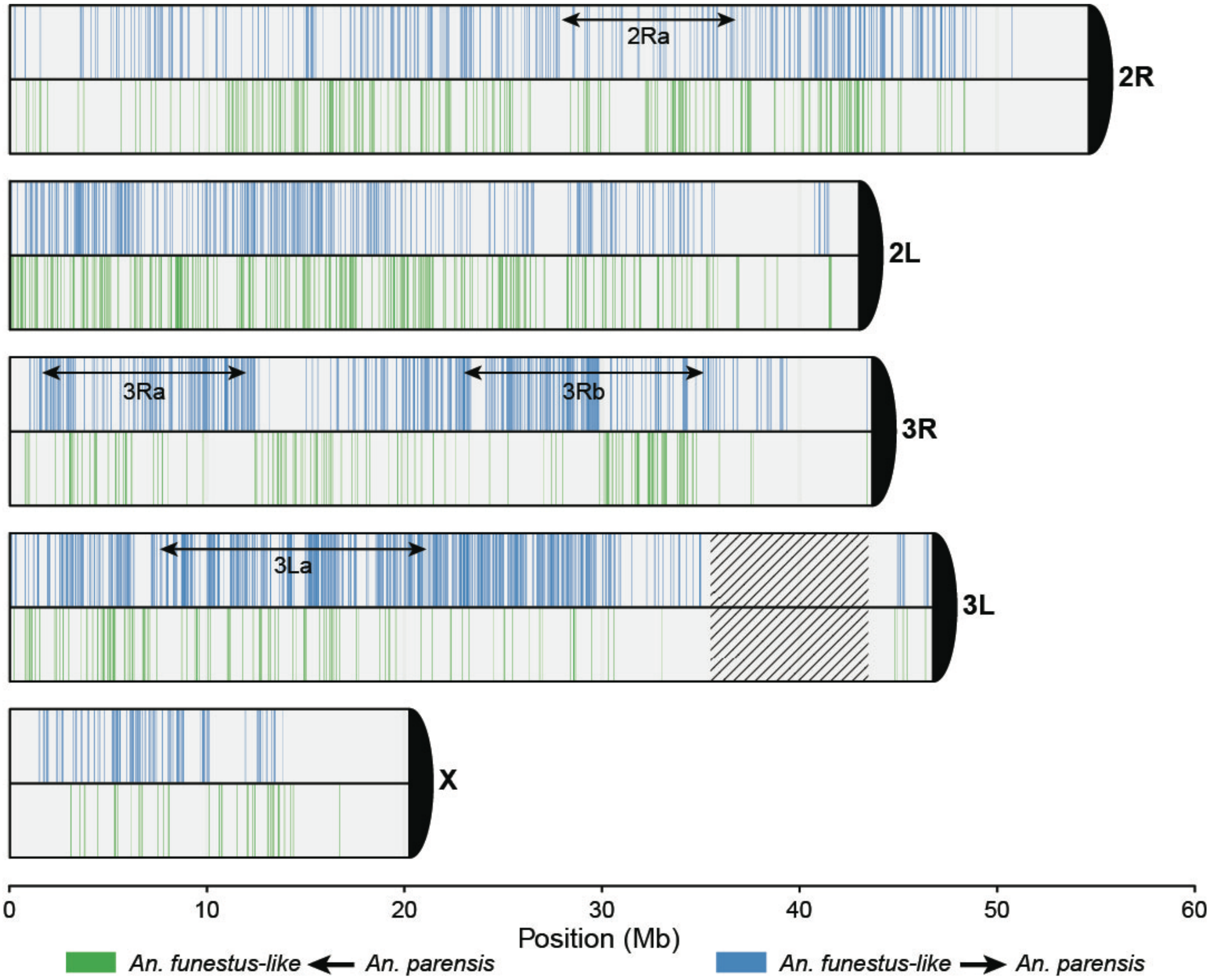

Fig. S19. ***Window-based estimates of introgression between An. funestus-like and An. parensis from FILET***. *Windows classified as introgressed at a probability of > 90% are shown on the vertical axis. Above the zero-line, blue represents regions that originated in An. funestus-like and introgressed into An. parensis. Below the zero-line, green represents genomic regions that originated in An. parensis and introgressed into An. funestus-like. Approximate locations of known inversions in An. funestus (3Ra, 3Rb, 3La, and 2Ra) are indicated by double-headed arrows. Centromeres are represented as black ¼ circles at the right of each chromosome arm. The hatched box represents masked region.*

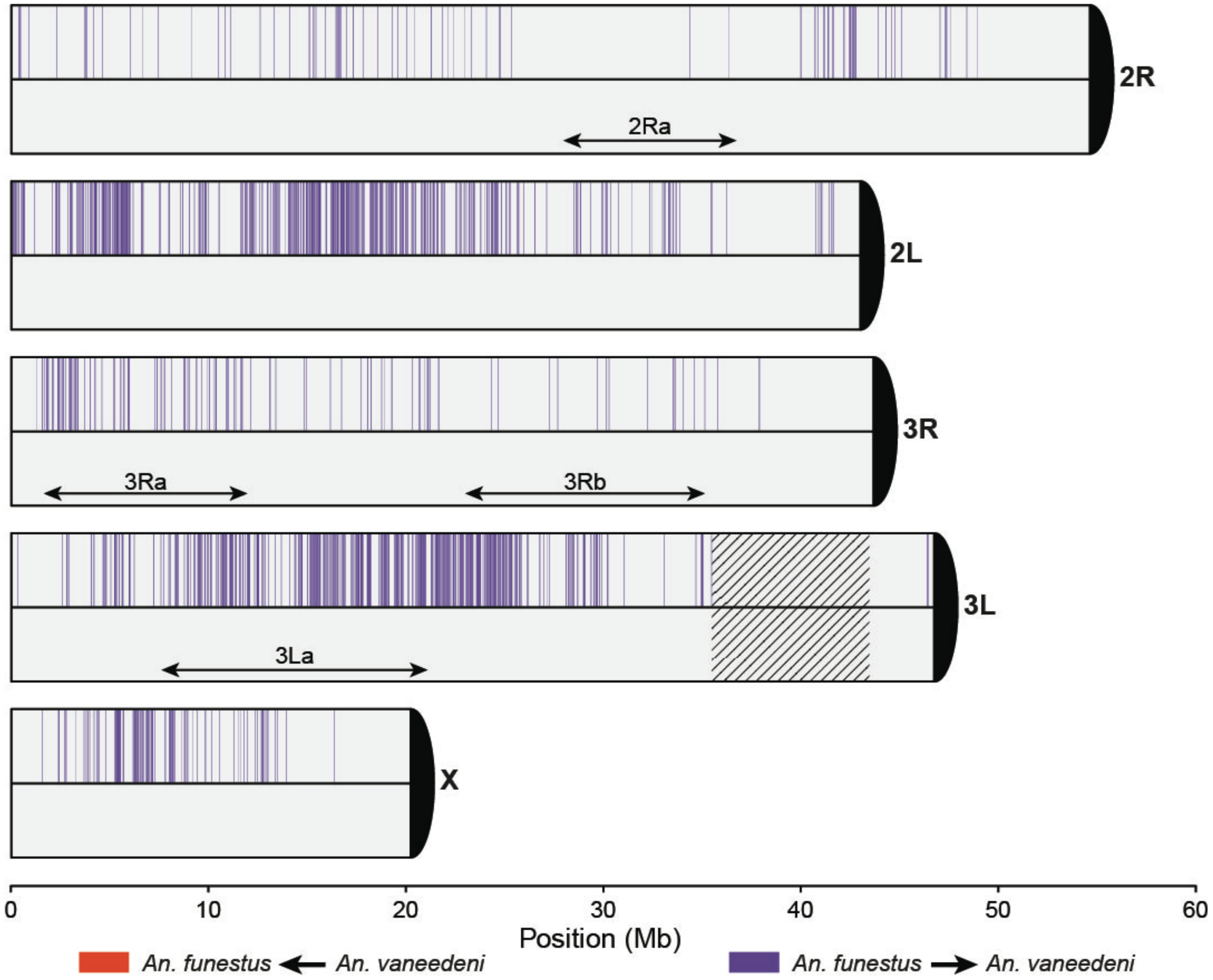

Fig. S20. ***Window-based estimates of introgression between An. funestus and An. vaneedeni from FILET.*** *Windows classified as introgressed at a probability of > 90% are shown on the vertical axis. Above the zero-line, purple represents regions that originated in An. funestus and introgressed into An. vaneedeni. No regions were classified with high confidence as having introgressed from An. vaneedeni to An. funestus. Approximate locations of known inversions in An. funestus (3Ra, 3Rb, 3La, and 2Ra) are indicated by double-headed arrows. Centromeres are represented as black ¼ circles at the right of each chromosome arm. The hatched box represents masked region.*

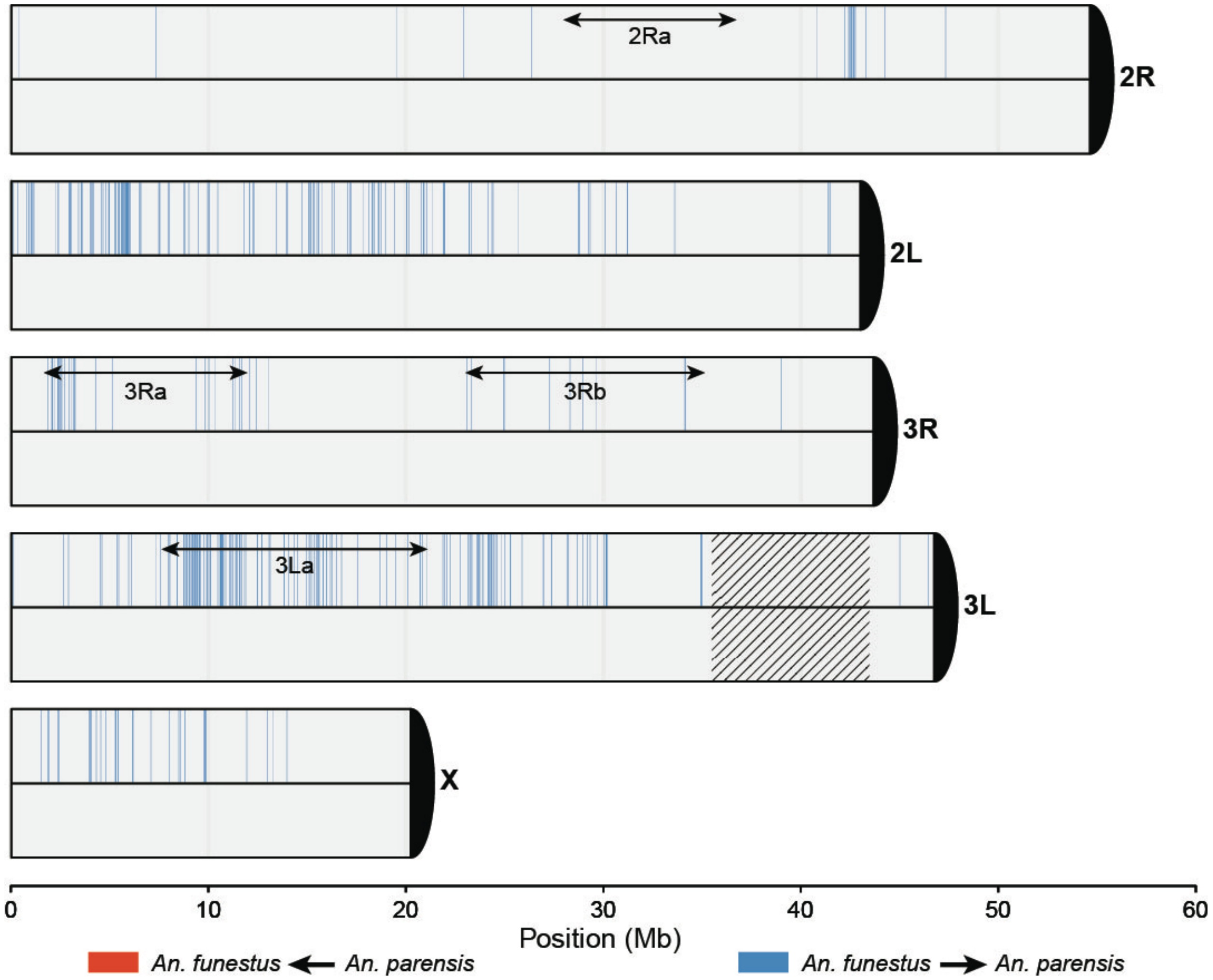

Fig. S21. ***Window-based estimates of introgression between An. funestus and An. parensis associated with Event A (Fig1D) from FILET.*** *Windows classified as introgressed at a probability of > 90% are shown on the vertical axis. Above the zero-line, blue represents regions that originated in An. funestus and introgressed into An. parensis. No regions were classified with high confidence as having introgressed from An. parensis to An. funestus. Approximate locations of known inversions in An. funestus (3Ra, 3Rb, 3La, and 2Ra) are indicated by double-headed arrows. Centromeres are represented as black ¼ circles at the right of each chromosome arm. The hatched box represents masked region.*

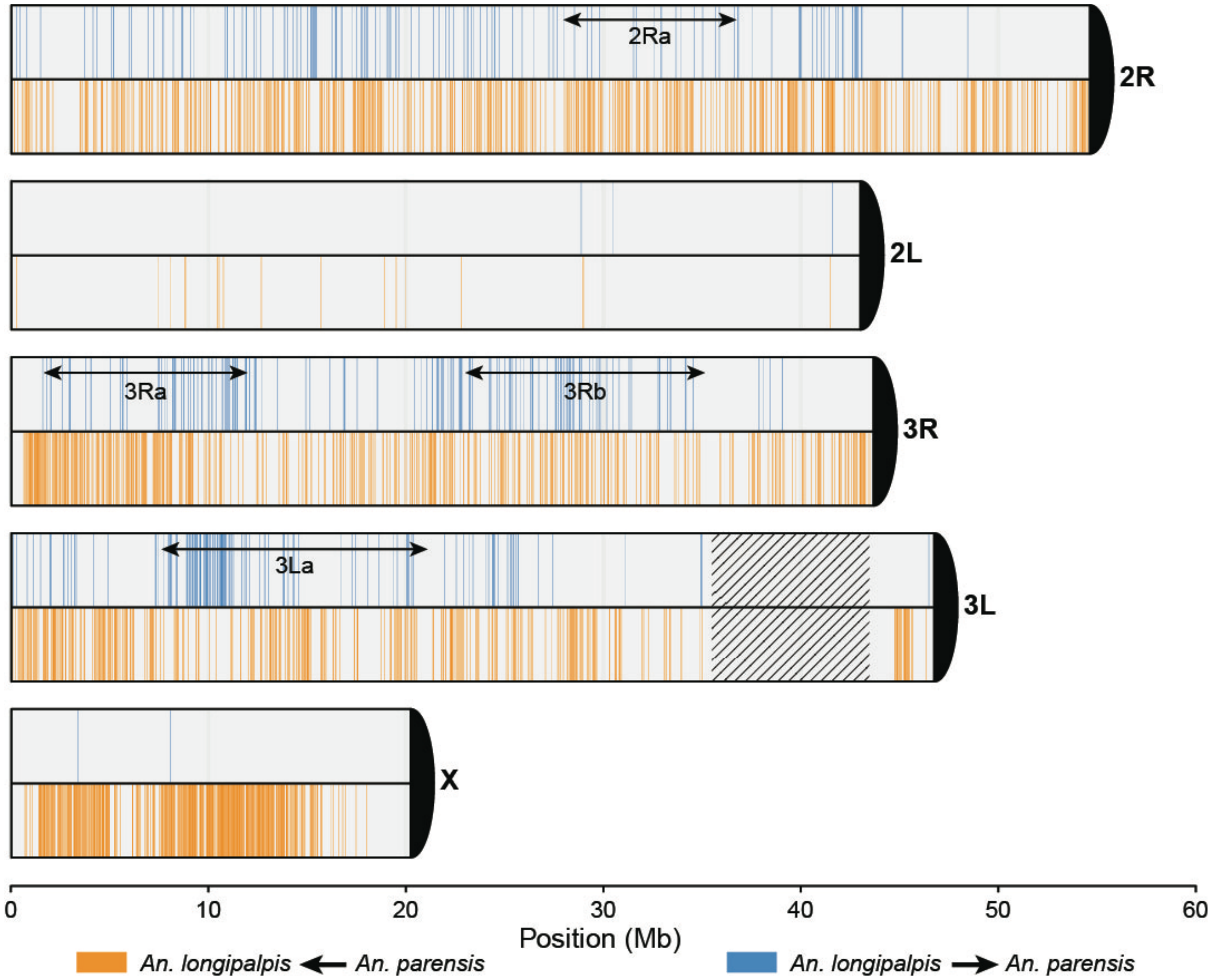

Fig. S22. *Window-based estimates of introgression between An. longipalpis C and An. parensis from FILET.* *Windows classified as introgressed at a probability of > 90% are shown on the vertical axis. Above the zero-line, blue represents regions that originated in An. longipalpis C and introgressed into An. parensis. Below the zero-line, orange represents genomic regions that originated in An. parensis and introgressed into An. longipalpis C. Approximate locations of known inversions in An. funestus (3Ra, 3Rb, 3La, and 2Ra) are indicated by double-headed arrows. Centromeres are represented as black ¼ circles at the right of each chromosome arm. The hatched box represents masked region.*

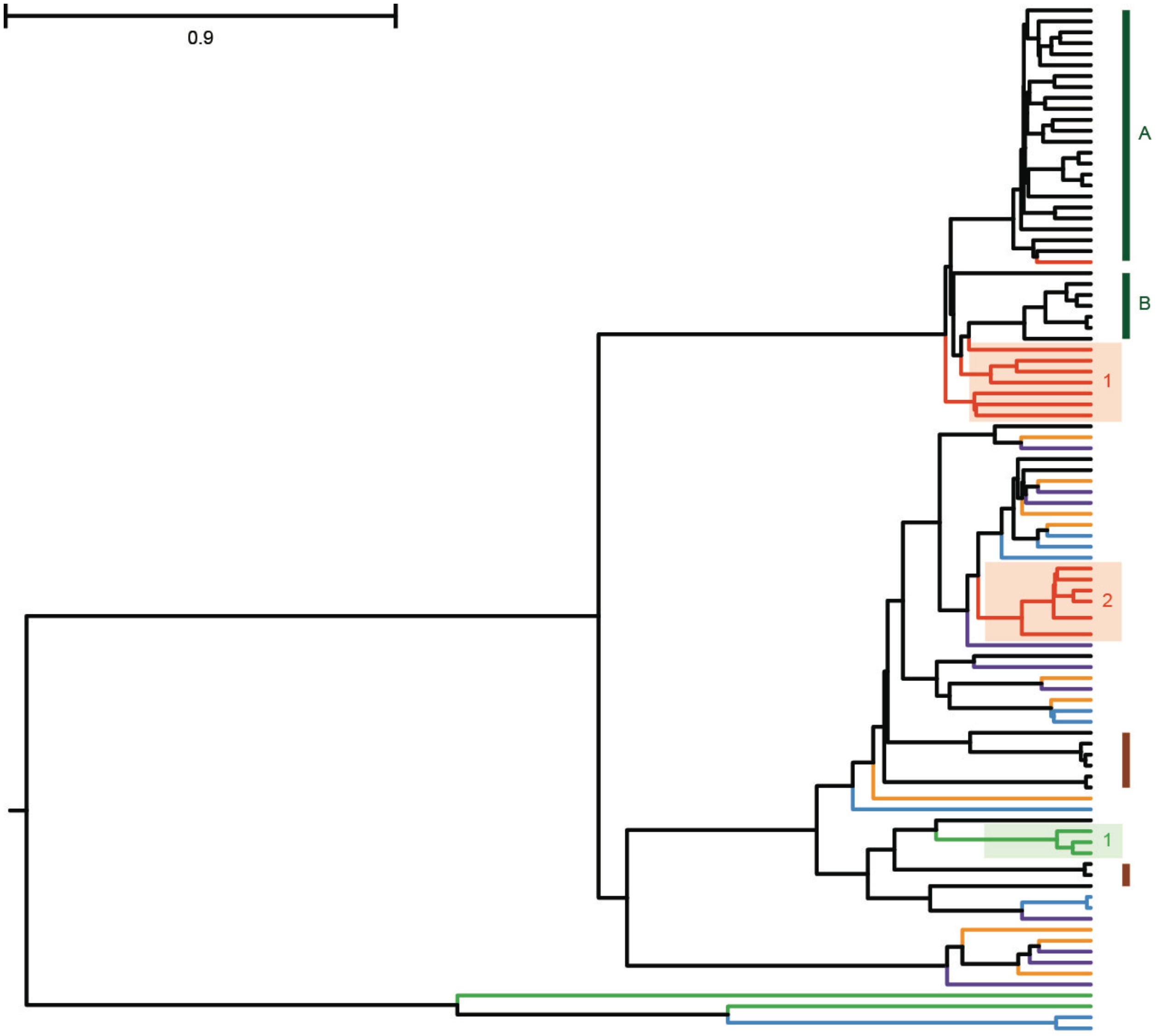

Fig. S23. ***Whole mitochondrial genome tree including samples from (55)***. *Samples from our study are represented by colored branches: An. funestus (red, highlighted clade 1 and clade 2 (18)), An. funestus-like (green), An. longipalpis C (orange), An. parensis (purple), and An. vaneedeni (blue). Samples from (55), all of which were morphologically identified as An. funestus, are represented with black tips. Lineages I and II from (18) are indicated by vertical dark green and brown bars, respectively. Subdivisions of Lineage I designated as A and B are labeled.*

Table S1. ***Sample information for 54 mosquito specimens sequenced for this project.***

Sample ID	Short form ID as found in figures	Species	Country	Province	Locality	District	Village	Year of collection	Count of homozygous for reference allele	Count of homozygous for nonreference allele	Count of heterzygous	Percent of the genome covered at >20x read depth
funestuscf.MalF105.7	Lik.Mal.01	*An. funestus-like*	Malawi	N/A	N/A	N/A	Karonga	2007	1214172	511941	1077477	0.945
funestuscf.MalF107.11 [1,2]	Lik.Mal.02	*An. funestus-like*	Malawi	N/A	N/A	N/A	Karonga	2007	1792031	1151	1097013	0.997
funestuscf.MalF99.4	Lik.Mal.03	*An. funestus-like*	Malawi	N/A	N/A	N/A	Karonga	2007	1156583	454012	953241	0.851
funestuscf.MalF90 [1]	Lik.Mal.04	*An. funestus-like*	Malawi	N/A	N/A	N/A	Karonga	2007	1081031	424245	849243	0.796
funestuscf.MalF98.2	Lik.Mal.05	*An. funestus-like*	Malawi	N/A	N/A	N/A	Karonga	2007	1156194	464438	995515	0.883
longipalpisC.Zam4	Lon.Zam.01	*An. longipalpis* type C	Zambia	N/A	N/A	Macha	Macha	2006	6982965	1130172	2204506	0.880
longipalpisC.Zam11	Lon.Zam.02	*An. longipalpis* type C	Zambia	N/A	N/A	Macha	Macha	2006	6996396	1137397	2202543	0.886
longipalpisC.Zam12	Lon.Zam.03	*An. longipalpis* type C	Zambia	N/A	N/A	Macha	Macha	2006	6932367	1098826	2203246	0.871
longipalpisC.Zam12852 [1]	Lon.Zam.04	*An. longipalpis* type C	Zambia	N/A	N/A	Nyimba	Nyimba	2011	7091123	1265678	2263339	0.948
longipalpisC.Zam13	Lon.Zam.05	*An. longipalpis* type C	Zambia	N/A	N/A	Macha	Macha	2006	6925829	1102419	2211052	0.867
longipalpisC.Zam14254 [1,2]	Lon.Zam.06	*An. longipalpis* type C	Zambia	N/A	N/A	Nyimba	Nyimba	2011	8997487	13605	1927434	0.990
longipalpisC.Zam15	Lon.Zam.07	*An. longipalpis* type C	Zambia	N/A	N/A	Macha	Macha	2006	6951693	1097660	2192472	0.872
longipalpisC.Zam16	Lon.Zam.08	*An. longipalpis* type C	Zambia	N/A	N/A	Macha	Macha	2006	6896149	1077284	2164702	0.847
longipalpisC.Zam12533	Lon.Zam.09	*An. longipalpis* type C	Zambia	N/A	N/A	Nyimba	Nyimba	2011	6840328	1033735	2107651	0.825
longipalpisC.Zam12634	Lon.Zam.10	*An. longipalpis* type C	Zambia	N/A	N/A	Nyimba	Nyimba	2011	6457688	896966	1837697	0.714
vaneedeni.KwaF659 [1,2]	Van.Kwa.01	*An. vaneedeni*	South Africa	Kwazulu Natal	Mamfene	N/A	N/A	22-Feb-2010	7772489	8718	1758263	0.993
vaneedeni.KwaF779 [1]	Van.Kwa.02	*An. vaneedeni*	South Africa	Kwazulu Natal	Ndumo	Mlube River	Homestead 2	17-Apr-2013	6216095	1040628	2048981	0.962
vaneedeni.KwaF773	Van.Kwa.03	*An. vaneedeni*	South Africa	Kwazulu Natal	Mseleni	Mseleni Str	Homestead 14	5-Feb-2013	6157572	913983	1892852	0.873
vaneedeni.KwaF774	Van.Kwa.04	*An. vaneedeni*	South Africa	Kwazulu Natal	Mamfene	Mtshazi Str	Homestead 157	15-Feb-2013	6172191	921331	1892118	0.879
vaneedeni.KwaF775	Van.Kwa.05	*An. vaneedeni*	South Africa	Kwazulu Natal	Mamfene	Mtshazi Str	Homestead 157	15-Feb-2013	6153035	906277	1892233	0.873
vaneedeni.KwaF780	Van.Kwa.06	*An. vaneedeni*	South Africa	Kwazulu Natal	Lower Umfolozi	Macekane Section 1	Vledi	20-Feb-2013	6179425	955460	1998039	0.912
vaneedeni.KwaF782	Van.Kwa.07	*An. vaneedeni*	South Africa	Kwazulu Natal	Ndumo, Section 7	Mlube River	Homestead 2	17-Apr-2013	6113049	915179	1927861	0.874
vaneedeni.KwaF783	Van.Kwa.08	*An. vaneedeni*	South Africa	Kwazulu Natal	Lower Umfolozi	Cinci Section 1	Umhlwathi str	18-Apr-2013	6197429	930099	1906751	0.893
vaneedeni.KwaF784	Van.Kwa.09	*An. vaneedeni*	South Africa	Kwazulu Natal	Lower Umfolozi	Cinci Section 1	Umhlwathi str	18-Apr-2013	6131708	901054	1865416	0.860
vaneedeni.KwaF786	Van.Kwa.10	*An. vaneedeni*	South Africa	Kwazulu Natal	Lower Umfolozi	Empangeni	Outside area	7-May-2013	6017734	914848	1724864	0.823
rivulorum.KwaF790 [1]	Riv.Kwa.01	*An. rivulorum*	South Africa	Kwazulu Natal	Makanis	Pongola River	Homestead 1	7-Apr-2013	709160	727307	1701593	0.983
rivulorum.KwaF794 [1,2]	Riv.Kwa.02	*An. rivulorum*	South Africa	Kwazulu Natal	Opansi	Ntelezi Str	Homestead 77	30-May-2013	1329311	139499	1680463	0.993
speciesA.Ken1007C2 [1,2]	SpA.Ken.01	*An. species A*	Kenya	N/A	N/A	Bigege	Bigege	2010	3015333	7535	794649	0.996
speciesA.Zam237C26	SpA.Zam.01	*An. species A*	Zambia	N/A	N/A	Nyimba	Nyimba	2011	350740	1614057	1858731	0.987
parensis.KwaF777 [1,2]	Par.Kwa.01	*An. parensis*	South Africa	Kwazulu Natal	Tete	Nhlozenkulu	Homestead 41	20-Mar-2013	4782425	5007	1541853	0.993
parensis.KwaF837 [1]	Par.Kwa.02	*An. parensis*	South Africa	Kwazulu Natal	Mamfene	N/A	N/A	20-Jan-2014	3650490	954120	1618958	0.973
parensis.KwaF761	Par.Kwa.03	*An. parensis*	South Africa	Kwazulu Natal	N/A	Makanis	Homestead 34	7-Jan-2013	3559761	835368	1729574	0.933
parensis.KwaF762	Par.Kwa.04	*An. parensis*	South Africa	Kwazulu Natal	N/A	Makanis	Homestead 34	7-Jan-2013	3550566	827360	1693993	0.917
parensis.KwaF766	Par.Kwa.05	*An. parensis*	South Africa	Kwazulu Natal	Tete	Mengu	Homestead 122	10-Jan-2013	3555343	833259	1735453	0.933
parensis.KwaF767	Par.Kwa.06	*An. parensis*	South Africa	Kwazulu Natal	Tete	Mengu	Homestead 122	10-Jan-2013	3558502	851731	1737142	0.941
parensis.KwaF768	Par.Kwa.07	*An. parensis*	South Africa	Kwazulu Natal	Tete	Mengu	Homestead 122	10-Jan-2013	3601223	900084	1628536	0.932
parensis.KwaF769	Par.Kwa.08	*An. parensis*	South Africa	Kwazulu Natal	Makanis	Clinic	Homestead 2	17-Jan-2013	3558957	834208	1713117	0.920
parensis.KwaF835	Par.Kwa.09	*An. parensis*	South Africa	Kwazulu Natal	Mamfene	N/A	N/A	29-Jan-2014	3542556	819059	1719326	0.916
parensis.KwaF851	Par.Kwa.10	*An. parensis*	South Africa	Kwazulu Natal	Mamfene	N/A	N/A	18-Feb-2014	3551956	833311	1716969	0.921
funestus.Ken4590C5 (I)	Fun.Ken.01	*An. funestus*	Kenya	N/A	N/A	Bigege	Bigege	2010	10460626	1364417	2676340	0.927
funestus.GhaF264 (I)	Fun.Gha.01	*An. funestus*	Ghana	N/A	N/A	N/A	Jimiso	2004	10336704	1408870	2474724	0.882
funestus.GhaF265 (I)	Fun.Gha.02	*An. funestus*	Ghana	N/A	N/A	N/A	Jimiso	2004	10191607	1346014	2382468	0.831
funestus.MozF220 (II) [1]	Fun.Moz.01	*An. funestus*	Mozambique	N/A	N/A	N/A	Chibuto	2007	11441907	1156840	2014283	0.952
funestus.MozF123 (II)	Fun.Moz.02	*An. funestus*	Mozambique	N/A	N/A	N/A	Chibuto	2007	11045550	963181	2162620	0.874
funestus.MozF260 (II)	Fun.Moz.03	*An. funestus*	Mozambique	N/A	N/A	N/A	Chibuto	2007	11225147	1035984	2150798	0.916
funestus.MozF29 (II)	Fun.Moz.04	*An. funestus*	Mozambique	N/A	N/A	N/A	Chibuto	2007	10971256	922779	2071095	0.851
funestus.MozF35 (II)	Fun.Moz.05	*An. funestus*	Mozambique	N/A	N/A	N/A	Chibuto	2007	10691070	907051	1879763	0.801
funestus.MozF804 (II)	Fun.Moz.06	*An. funestus*	Mozambique	N/A	N/A	N/A	Tavira	24-Nov-2014	11034575	932591	2032964	0.844
funestus.TanF561 (I)	Fun.Tan.01	*An. funestus*	Tanzania	N/A	N/A	N/A	Geita	13-May-2005	10541437	1105238	2478131	0.864
funestus.TanF601 (I)	Fun.Tan.02	*An. funestus*	Tanzania	N/A	N/A	N/A	Geita	13-May-2005	10606213	1085007	2324036	0.845
funestus.UgaF399 (I)	Fun.Uga.01	*An. funestus*	Uganda	N/A	N/A	N/A	Tororo Amoni	2001	10409109	1429343	2473409	0.894
funestus.UgaF401 (I)	Fun.Uga.02	*An. funestus*	Uganda	N/A	N/A	N/A	Tororo Amoni	2001	10401066	1361819	2552087	0.896
funestus.UgaF403 (I)	Fun.Uga.03	*An. funestus*	Uganda	N/A	N/A	N/A	Tororo Amoni	2001	10178671	1243975	2475870	0.831
funestus.Zam281C17 (I)	Fun.Zam.01	*An. funestus*	Zambia	N/A	N/A	Nyimba	Nyimba	2011	11062617	1277171	2194615	0.937

[1]Sequenced as described in S2 for *de novo* genome assembly. [2]Selected for final *de novo* assembly. (I) mitochondrial clade 1, (II) mitochondrial clade 2

Table S2. *Final assembly statistics.* *For each species assembly the assigned chromosome and scaffold lengths are listed in base pairs. Multiple scaffolds assigned to the same chromosome indicate fragmentation.*

Species	CHR	SCAFFOLD		SCAFFOLD		SCAFFOLD		SCAFFOLD		SCAFFOLD		SCAFFOLD	
		**Name**	**Size (bp)**	**Name**	**Size (bp)**	**Name**	**Size (bp)**	**Name**	**Size (bp)**	**Name**	**Size (bp)**	**Name**	**Size (bp)**
An. funestus-like	X	X	20,187,332										
	3R	3R	43,687,904										
	3L	3L	34,205,828	3L.1	3,015,323								
	2R	2R	39,085,082	2R.1	1,223,783	2R.2	708,654						
	2L	2L	44,372,129	2L.1	14,676,318								
	mtDNA		15,195										
An. longipalpis C	X	X	22,423,081										
	3R	3R	49,681,555										
	3L	3L	36,616,011	3L.1	3,251,674								
	2R	2R	18,736,924	2R.1	40,343,036								
	2L	2L	49,924,677										
	mtDNA		15,353										
An. parensis	X	X	20,062,383										
	3R	3R	45,199,288										
	3L	3L	43,336,102	3L.1	7,103,730								
	2R	2R	34,068,625	2R.1	17,236,664	2R.2	4,166,336	2R.3	333,518				
	2L	2L	30,688,485	2L.1	14,776,828								
	mtDNA		15,409										
An. vaneedeni	X	X	21,833,418										
	3R	3R	47,560,645										
	3L	3L	43,164,299	3L.1	7,277,665								
	2R	2R	57,364,019	2R.1	271,663								
	2L	2L	47,023,152	2L.1	1,239,921								
	mtDNA		15,353										
An. rivulorum	X	X	16,059,766	X.1	1,536,380	X.2	1,660,424						
	3R	3RL	45,054,358										
	3L	3L.1	25,273,932	3L.2	7,130,752	3L.3	4,181,684						
	2R	2RL	33,018,472	2R.1	29,847,557	2R.2	3,628,986	2R.3	3,289,573	2R.4	698647		
	2L	2L.1	28,033,306	2L.2	304,522								
	mtDNA		15,358										
An. species A	X	X	16,987,791	X.1	6,452,217								
	3R	3R	41,861,234										
	3L	3L	20,442,961	3L.1	7,124,036	3L.2	2,996,056	3L.3	192,460				
	2R	2R	29,633,918	2R.1	16,596,037	2R.2	5,585,933	2R.3	1,048,787	2R.4	124557	2R.5	222154
	2L	2L	27,609,615	2L.1	16,003,870								
	mtDNA		15,358										

Table S3. ***Whole genome alignment statistics for each AFC species along each chromosome arm.*** *Total aligned bases are listed for each chromosome arm with the percent aligned to the An. funestus reference genome in parentheses*.

Species	2R	2L	3R	3L	X
An. funestus-like	42,055,471 (0.77)	32,399,296 (0.73)	33,085,944 (0.76)	25,157,859 (0.54)	15,707,801 (0.78)
An. vaneedeni	42,055,471 (0.77)	32,399,296 (0.73)	33,085,944 (0.76)	24,691,972 (0.53)	15,506,419 (0.77)
An. longipalpis C	40,963,121 (0.75)	31,511,644 (0.71)	32,215,262 (0.74)	23,294,314 (0.50)	15,305,037 (0.76)
An. parensis	41,509,296 (0.76)	31,955,470 (0.72)	32,650,603 (0.75)	24,226,086 (0.52)	15,506,419 (0.77)
An. rivulorum	29,493,447 (0.54)	23,966,602 (0.54)	23,073,093 (0.53)	16,771,906 (0.36)	8,860,811 (0.44)

Table S4. ***Total SNPs for each species and population.*** *Total count of single nucleotide polymorphisms for each species and population sample, based on sites with no missing data aligned to the con-specific reference genome, with minor allele frequency > 5%.*

Species	Country	Individuals	SNPs
An. funestus	All	15	10,722,216
	Ghana	2	4,914,943
	Uganda	3	6,182,436
	Mozambique	6	4,986,376
	Tanzania	2	4,515,652
	Kenya	1	2,676,340
	Zambia	1	2,194,615
An. funestus-like	Malawi	5	2,108,997
An. longipalpis C	Zambia	10	7,954,846
An. parensis	South Africa	10	5,393,623
An. vaneedeni	South Africa	10	7,436,725
An. species A	Kenya	1	788,030
	Zambia	1	3,463,088
An. rivulorum	South Africa	2	2,652,487

Table S5. ***Divergence times in generations among An. funestus populations.*** *Estimates from (36) on the upper triangle and estimates from MSMC2 on the lower triangle. Estimates can be converted to years by dividing by 11 generations per year.*

Pops	Ghana	Kenya	Mozambique	Tanzania	Uganda	Zambia
**Ghana**	**0**	9,867	15,879	18,458	4,790	24,093
**Kenya**	7,000	**0**	54,246	154,305	7,517	NA
**Mozambique**	2,900	70,000	**0**	190	5,817	23,076
**Tanzania**	3,900	15,000	7,000	**0**	5,735	3,517
**Uganda**	2,700	NA	5,900	2,800	**0**	5,740
**Zambia**	28,000	NA	6,500	9,800	6,900	**0**

Table S6. ***D* statistics for each triplet (X,Y,Z) with *An. rivulorum* as outgroup (W).** *If the Z-score is negative, then gene flow is inferred between X and Y. If the Z-score is positive, then gene flow is inferred between X and Z. Abbreviations: An. funestus (Fun), An. funestus-like (Lik), An. longipalpis C (Lon), An. parensis (Par), An. vaneedeni (Van), and An. rivulorum (Riv).*

W	X	Y	Z	*D*	SE	Z-score
Riv	Par	Lon	Lik	-0.449	0.049	-9.108
Riv	Van	Par	Lik	0.117	0.05	2.328
Riv	Lon	Par	Lik	-0.418	0.048	-8.691
Riv	Lik	Van	Par	-0.247	0.038	-6.502
Riv	Lik	Par	Lon	0.038	0.03	1.276
Riv	Par	Van	Lik	-0.133	0.03	-4.393
Riv	Fun	Lik	Par	-0.467	0.046	-10.083
Riv	Par	Fun	Lik	0.093	0.025	3.769
Riv	Lik	Fun	Lon	-0.369	0.067	-5.489
Riv	Fun	Lik	Van	-0.3	0.041	-7.305
Riv	Lik	Fun	Van	-0.216	0.057	-3.797
Riv	Fun	Lik	Lon	-0.451	0.052	-8.736
Riv	Lon	Van	Lik	-0.236	0.018	-13.009
Riv	Lik	Van	Lon	-0.235	0.037	-6.405
Riv	Van	Lon	Lik	-0.002	0.055	-0.028
Riv	Lon	Van	Par	0.256	0.075	3.428
Riv	Fun	Lon	Van	0.251	0.038	6.63
Riv	Van	Lon	Fun	-0.054	0.064	-0.841
Riv	Van	Par	Lon	0.154	0.016	9.93
Riv	Lon	Par	Fun	-0.464	0.041	-11.348
Riv	Par	Van	Fun	-0.198	0.034	-5.753
Riv	Van	Fun	Lik	0.09	0.02	4.613
Riv	Par	Lon	Fun	-0.49	0.039	-12.651
Riv	Lon	Fun	Lik	0.098	0.02	4.933
Riv	Fun	Par	Lon	0.034	0.029	1.185
Riv	Fun	Van	Par	-0.259	0.035	-7.403
Riv	Lik	Fun	Par	-0.391	0.033	-11.847
Riv	Van	Par	Fun	0.064	0.059	1.097
Riv	Lon	Van	Fun	-0.301	0.023	-12.809
Riv	Par	Lon	Van	-0.395	0.071	-5.589

Table S7. ***Pairwise divergence (d_{XY}) and estimated divergence times.*** *d_{XY} values in the lower triangle reflect the mean for 10-kb windows along the genome. Divergence time in generations in the upper triangle assumes a mutation rate of 2.8 x 10^{-9}, and an ancestral effective population size of 1,400,000. Abbreviations: An. funestus (Fun), An. funestus-like (Lik), An. longipalpis C (Lon), An. parensis (Par), and An. vaneedeni (Van).*

Species	Fun	Lik	Van	Lon	Par
**Fun**	0	1,599,810	2,226,000	3,001,983	3,090397
**Lik**	0.01798	0	2,215,241	2,820,259	3,061,466
**Van**	0.02161	0.02154	0	2,679,172	2,962,172
**Lon**	0.02611	0.02505	0.02424	0	1,742,276
**Par**	0.02662	0.02645	0.02588	0.01881	0

Table S8. *Priors for coalescent simulations.* *Priors were defined using estimates of divergence times (Table S7). Population histories were based on the MSMC2 results with initial population sizes inferred by the median of the population size in the last epoch. Notation of U~[Lower, Upper] refers to uniform priors with range in brackets. Abbreviations: An. funestus (Fun), An. funestus-like (Lik), An. longipalpis C (Lon), An. parensis (Par), and An. vaneedeni (Van).*

Species	Initial population sizes
An. funestus-like	20,993
An. funestus	178,924
An. vaneedeni	117,587
An. longipalpis C	92,530
An. parensis	70,861

Parameter	tree *i.*	tree *iii.*	tree *vii.*
Div Lik/Fun	U~[450000, 1500000]	U~[600000, 1500000]	U~[450000, 1500000]
Div Lon/Par	U~[800000, 1700000]	U~[500000, 1700000]	N/A
Div Van/Lon	N/A	N/A	U~[600000, 1000000]
Div (Van,Lon)/Par	N/A	U~[1200000, 1400000]	U~[1000000, 2900000]
Div (Fun,Lik)/Van	U~[1200000, 2200000]	N/A	N/A
Div (Fun,Lik)/Par	U~[2500000, 6500000]	U~[2500000, 6500000]	U~[2200000, 6500000]
Adx Time 1	U~[10000, 300000]	U~[160000, 240000]	U~[160000, 240000]
Adx Time 2	U~[160000, 240000]	U~[800000, 1000000]	U~[800000, 1000000]
Adx Time 3	U~[800000, 1500000]	U~[800000, 1500000]	U~[800000, 1500000]

Table S9. ***Confusion matrix for classification between the three models in Fig. 3.*** *Prior error rate was 0.0335 for 1,000 decision trees.*

**tree**	**tree *i*.**	**tree *iii*.**	**tree *vii*.**	**Class error**
**tree i.**	93,920	559	5,365	0.059333
**tree iii.**	447	99,357	72	0.005196
**tree vii.**	3,573	45	96,231	0.036235

Table S10. ***Model classification results where total votes = 1,000.***

Chromosome	Selected model	# votes tree *i.*	# votes tree *iii.*	# votes tree *vii.*	Posterior probability
2L	tree i.	425	175	400	0.661
2R	tree vii.	294	29	677	0.690
3L	tree vii.	304	44	652	0.699
3R	tree vii.	288	38	674	0.684

Table S11. ***Estimates of model parameters under tree vii using approximate Bayesian computation***. *Times are in generations with 95% credible intervals in parentheses. Abbreviations: An. funestus (Fun), An. funestus-like (Lik), An. longipalpis C (Lon), An. parensis (Par), and An. vaneedeni (Van).*

**Species**	**Effective population size**		
Fun	447,773		
Lik	68,084		
Van	94,333		
Lon	94,539		
Par	87,131		
Riv	105,265		
		**Divergence time (gens)**	
(Fun,Lik)	301,435	415,648 (336,884-496,613)	
(Lon,Van)	741,890	762,677 (684,603-864,727)	
(Lon,Van), Par	867,631	1,049,380 (997,056-1,099,797)	
((Lon,Van),Par),(Fun,Lik)	1,090,732	2,375,194 (2,340,050-2,440,225)	
(AFC), Riv	N/A	26,625,000 (21,332,142-40,328,571)	
		**Introgression time (gens)**	**Introgression proportion**
Par → Fun [Event C, Fig. 1D]	165,020	146,273 (130,530-162,123)	0.228 (0.111-0.385)
(Fun,Lik) → Van	404,124	860,897 (752,858-953,997)	0.329 (0.295-0.350)
(Fun,Lik) → Par [Event A, Fig. 1D]	404,124	860,355 (753,045-949,668)	0.221 (0.171-0.272)

Table S12. ***Confusion matrix for classification of introgression direction in FILET****. The confusion matrix was constructed by leaving out 10,000 feature vectors from the 60,000 feature-vector training set. The trained classifier was then tested on the left-out training data. The trained model had a higher probability of classifying introgression as no introgression (false negatives) rather than the wrong direction. Probability of true positives are along the diagonal and highlighted in blue, where darker shades are associated with higher probabilities. Abbreviations: An. funestus (Fun), An. funestus-like (Lik), An. parensis (Par), An. vaneedeni (Van); Introg, introgression.*

**Fun ↔ Par**	**No Introg**	**Par → Fun**	**Fun → Par**
**No Introg**	1.000	0.000	0.000
**Par → Fun**	0.091	0.900	0.009
**Fun → Par**	0.061	0.010	0.930

**Lik ↔ Van**	**No Introg**	**Van → Lik**	**Lik → Van**
**No Introg**	0.994	0.002	0.004
**Van → Lik**	0.406	0.548	0.047
**Lik → Van**	0.163	0.033	0.804

**Lik ↔ Par**	**No Introg**	**Par → Lik**	**Lik → Par**
**No Introg**	1.000	0.000	0.000
**Par → Lik**	0.329	0.677	0.006
**Lik → Par**	0.036	0.007	0.958

Table S13. *Introgressed DNA between species pairs (in Megabases) as classified using FILET.* *Introgression events are assigned a letter (A-I); events A-C are shown in Fig. 1D. Introgression is broken down by direction, and by autosome versus X chromosome. The proportions are based on the total accessible base pairs, mean = 158.61. Abbreviations: An. funestus (Fun), An. funestus-like (Lik), An. longipalpis C (Lon), An. parensis (Par), and An. vaneedeni (Van).*

			Direction (Mb)		Total (Mb)		Proportion	
**Event**	**Species 1**	**Species 2**	**1→2**	**2→1**	**Autosomes**	**X**	**Autosomes**	**X**
A	Lik	Par	11.65	3.39	14.13	0.90	0.09	0.05
A	Fun	Par	5.92	0.00	4.10	1.83	0.03	0.10
B	Lik	Van	20.25	7.77	23.35	4.67	0.15	0.26
B	Fun	Van	16.71	0.00	13.57	3.13	0.09	0.18
C	Fun	Par	1.07	31.61	23.91	9.56	0.15	0.53
D	Lon	Par	5.22	27.16	23.58	8.80	0.15	0.49
E	Van	Par	0.33	2.77	3.02	0.08	0.02	0.00
F	Fun	Lon	9.66	0.00	5.28	4.38	0.03	0.25
G	Van	Lon	0.62	0.85	1.39	0.08	0.01	0.00
H	Fun	Lik	0.97	0.87	1.38	0.47	0.01	0.03
I	Lik	Lon	0.00	4.55	3.29	1.26	0.02	0.07

Table S14. ***Introgressed DNA between species pairs based on triplet topologies evaluated using QuIBL.*** *For each event (corresponding to Table S13) we provide the total number of base pairs (in Mb) for the autosomes and X chromosome, and the corresponding proportions based on the accessible genome (autosomes = 110 Mb; X = 12 Mb). Some events are shown multiple times because the introgression involved ancestral lineages. Note that events G and H are not shown, as QuIBL cannot detect introgression between sister taxa.*

Event	Species 1	Species 2	Outgroup	Autosome (Mb)	X (Mb)	Proportion Autosome	Proportion X
**A**	Fun	Par	Van	6.56	0.61 (ns)[1]	0.059	0.040
**A**	Fun	Par	Lon	1.64	0.16 (ns)[1]	0.015	0.012
**A**	Lik	Par	Fun	12.12	1.2 (ns)[1]	0.110	0.095
**A**	Lik	Par	Van	6.12	0.6 (ns)[1]	0.056	0.047
**A**	Lik	Par	Lon	1.84	0.16 (ns)[1]	0.017	0.012
**B**	Fun	Van	Par	55.69	4.15	0.505	0.331
**B**	Fun	Van	Lon	55.07	4.15	0.500	0.331
**B**	Fun	Van	Lik	34.23	3.95 (ns)[1]	0.310	0.331
**B**	Lik	Van	Par	52.48	3.95	0.476	0.315
**B**	Lik	Van	Lon	51.83	3.95	0.470	0.315
**B**	Lik	Van	Fun	17.26	1.42 (ns)[1]	0.157	0.113
**C**	Fun	Par	Lik	26.21	2.43 (ns)[1]	0.238	0.194
**D**	Lon	Par	Van	42.40	9.75	0.385	0.780
**E**	Van	Par	Lon	3.30	0.74 (ns)[1]	0.030	0.060
**F**	Fun	Lon	Lik	9.26	2.42 (ns)[1]	0.084	0.190
**F**	Fun	Lon	Van	6.33	0.58 (ns)[1]	0.057	0.046
**F**	Fun	Lon	Par	2.18	0.05 (ns)[1]	0.020	0.003
**I**	Lik	Lon	Fun	12.18	1.21 (ns)[1]	0.110	0.096
**I**	Lik	Lon	Van	5.87	0.57 (ns)[1]	0.053	0.045
**I**	Lik	Lon	Par	2.18	0.06 (ns)[1]	0.020	0.005

[1]ns, QuIBL detected introgression or ILS for the number of Mb indicated, but could not statistically distinguish between the two, due to low numbers of counts on the X chromosome.

SI References

1. C. Garros, R. E. Harbach, S. Manguin, Morphological assessment and molecular phylogenetics of the Funestus and Minimus Groups of *Anopheles* (*Cellia*). *J. Med. Entomol.* **42**, 522-536 (2005).
2. J. Stevenson *et al.*, Novel vectors of malaria parasites in the western highlands of Kenya. *Emerg. Infect. Dis.* **18**, 1547-1549 (2012).
3. N. F. Lobo *et al.*, Unexpected diversity of *Anopheles* species in Eastern Zambia: implications for evaluating vector behavior and interventions using molecular tools. *Sci. Rep.* **5**, 17952 (2015).
4. B. St Laurent *et al.*, Molecular characterization reveals diverse and unknown malaria vectors in the western Kenyan Highlands. *Am. J. Trop. Med. Hyg.* **94**, 327-335 (2016).
5. H. Chen, M. Rangasamy, S. Y. Tan, H. Wang, B. D. Siegfried, Evaluation of five methods for total DNA extraction from western corn rootworm beetles. *PLoS One* **5**, e11963 (2010).
6. L. Koekemoer, M. M. Weeto, L. Kamau, R. H. Hunt, M. Coetzee, A cocktail polymerase chain reaction (PCR) assay to identify members of the *Anopheles funestus* (Diptera: Culicidae) group. *Am. J. Trop. Med. Hyg.* **66**, 804-811 (2002).
7. K. S. Choi, M. Coetzee, L. L. Koekemoer, Simultaneous identification of the *Anopheles funestus* group and *Anopheles longipalpis* type C by PCR-RFLP. *Malar. J.* **9**, 316 (2010).
8. H. Li, R. Durbin, Fast and accurate short read alignment with Burrows-Wheeler transform. *Bioinformatics* **25**, 1754-1760 (2009).
9. B. J. Clavijo *et al.*, W2RAP: a pipeline for high quality, robust assemblies of large complex genomes from short read data. http://dx.doi.org/https://doi.org/10.1101/110999.
10. F. A. Simão, R. M. Waterhouse, P. Ioannidis, E. V. Kriventseva, E. M. Zdobnov, BUSCO: assessing genome assembly and annotation completeness with single-copy orthologs. *Bioinformatics* **31**, 3210–3212 (2015).
11. L. P. Pryszcz, T. Gabaldon, Redundans: an assembly pipeline for highly heterozygous genomes. *Nucleic Acids Res.* **44**, e113 (2016).
12. J. Ghurye *et al.*, A chromosome-scale assembly of the major African malaria vector *Anopheles funestus*. *GigaScience* **8** (2019).
13. B. Paten *et al.*, Cactus graphs for genome comparisons. *J. Comput. Biol.* **18**, 469-481 (2011).
14. B. Paten *et al.*, Cactus: Algorithms for genome multiple sequence alignment. *Genome Res.* **21**, 1512-1528 (2011).
15. M. Kolmogorov, B. Raney, B. Paten, S. Pham, Ragout-a reference-assisted assembly tool for bacterial genomes. *Bioinformatics* **30**, i302-309 (2014).
16. M. Tarailo-Graovac, N. Chen, Using RepeatMasker to identify repetitive elements in genomic sequences. *Curr Protoc Bioinformatics* **Chapter 4**, Unit 4 10 (2009).
17. D. E. Neafsey *et al.*, Highly evolvable malaria vectors: the genomes of 16 *Anopheles* mosquitoes. *Science* **347**, 1258522 (2015).
18. A. P. Michel *et al.*, Rangewide population genetic structure of the African malaria vector *Anopheles funestus*. *Mol. Ecol.* **14**, 4235-4248 (2005).

19. J. Krzywinski, O. G. Grushko, N. J. Besansky, Analysis of the complete mitochondrial DNA from *Anopheles funestus*: an improved dipteran mitochondrial genome annotation and a temporal dimension of mosquito evolution. *Mol. Phylogenet. Evol.* **39**, 417-423 (2006).
20. A. Steele, M. C. Fontaine, A. Martin, S. J. Emrich, Tools and Methods from the Anopheles 16 Genome Project.
21. R. Bouckaert *et al.*, BEAST 2: a software platform for Bayesian evolutionary analysis. *PLoS Comput. Biol.* **10**, e1003537 (2014).
22. G. Hickey, B. Paten, D. Earl, D. Zerbino, D. Haussler, HAL: a hierarchical format for storing and analyzing multiple genome alignments. *Bioinformatics* **29**, 1341-1342 (2013).
23. M. Blanchette *et al.*, Aligning multiple genomic sequences with the threaded blockset aligner. *Genome Res.* **14**, 708-715 (2004).
24. J. Y. Dutheil, S. Gaillard, E. H. Stukenbrock, MafFilter: a highly flexible and extensible multiple genome alignment files processor. *BMC Genomics* **15**, 53 (2014).
25. H. Li, A statistical framework for SNP calling, mutation discovery, association mapping and population genetical parameter estimation from sequencing data. *Bioinformatics* **27**, 2987-2993 (2011).
26. A. McKenna *et al.*, The Genome Analysis Toolkit: a MapReduce framework for analyzing next-generation DNA sequencing data. *Genome Res.* **20**, 1297-1303 (2010).
27. A. Miles, N. J. Harding, scikit-allel: A Python package for exploring and analysing genetic variation data. http://github.com/cggh/scikit-allel. http://dx.doi.org/10.5281/zenodo.597309.
28. O. Delaneau, J. F. Zagury, J. Marchini, Improved whole-chromosome phasing for disease and population genetic studies. *Nat Methods* **10**, 5-6 (2013).
29. A. S. Malaspinas *et al.*, A genomic history of Aboriginal Australia. *Nature* **538**, 207-214 (2016).
30. P. D. Keightley, R. W. Ness, D. L. Halligan, P. R. Haddrill, Estimation of the spontaneous mutation rate per nucleotide site in a *Drosophila melanogaster* full-sib family. *Genetics* **196**, 313-320 (2014).
31. M. Nei, W. H. Li, Mathematical model for studying genetic variation in terms of restriction endonucleases. *Proc. Natl. Acad. Sci. U. S. A.* **76**, 5269-5273 (1979).
32. F. Tajima, Statistical method for testing the neutral mutation hypothesis by DNA polymorphism. *Genetics* **123**, 585-595 (1989).
33. P. D. Keightley, B. C. Jackson, Inferring the Probability of the Derived vs. the Ancestral Allelic State at a Polymorphic Site. *Genetics* **209**, 897-906 (2018).
34. P. Hermann, A. Heissl, I. Tiemann-Boege, A. Futschik, LDJump: Estimating variable recombination rates from population genetic data. *Mol. Ecol. Resour.* **19**, 623-638 (2019).
35. J. A. Kamm, J. P. Spence, J. Chan, Y. S. Song, Two-Locus Likelihoods Under Variable Population Size and Fine-Scale Recombination Rate Estimation. *Genetics* **203**, 1381-1399 (2016).

36. C. Theunert, M. Slatkin, Estimation of population divergence times from SNP data and a test for treeness. BioRxiv. http://dx.doi.org/https://doi.org/10.1101/281881.
37. K. Katoh, K. Misawa, K. Kuma, T. Miyata, MAFFT: a novel method for rapid multiple sequence alignment based on fast Fourier transform. *Nucleic Acids Res.* **30**, 3059-3066 (2002).
38. T. Jombart, adegenet: a R package for the multivariate analysis of genetic markers. *Bioinformatics* **24**, 1403-1405 (2008).
39. T. Jombart, I. Ahmed, adegenet 1.3-1: new tools for the analysis of genome-wide SNP data. *Bioinformatics* **27**, 3070-3071 (2011).
40. E. Paradis, J. Claude, K. Strimmer, APE: Analyses of Phylogenetics and Evolution in R language. *Bioinformatics* **20**, 289-290 (2004).
41. Z. N. Kamvar, J. F. Tabima, N. J. Grunwald, Poppr: an R package for genetic analysis of populations with clonal, partially clonal, and/or sexual reproduction. *PeerJ* **2**, e281 (2014).
42. B. J. Knaus, N. J. Grunwald, vcfr: a package to manipulate and visualize variant call format data in R. *Mol. Ecol. Resour.* **17**, 44-53 (2017).
43. S. Guindon, F. Delsuc, J. F. Dufayard, O. Gascuel, Estimating maximum likelihood phylogenies with PhyML. *Methods Mol. Biol.* **537**, 113-137 (2009).
44. K. Leppala, S. V. Nielsen, T. Mailund, admixturegraph: an R package for admixture graph manipulation and fitting. *Bioinformatics* **33**, 1738-1740 (2017).
45. R. E. Green *et al.*, A draft sequence of the Neandertal genome. *Science* **328**, 710-722 (2010).
46. N. Patterson *et al.*, Ancient admixture in human history. *Genetics* **192**, 1065-1093 (2012).
47. P. Pudlo *et al.*, Reliable ABC model choice via random forests. *Bioinformatics* **32**, 859-866 (2016).
48. M. A. Beaumont, Approximate Bayesian Computation in Evolution and Ecology. *Annu. Rev. Ecol. Evol. Syst.* **41**, 379-406 (2010).
49. M. W. Hahn, *Molecular Population Genetics* (Sinauer Associates/Oxford University Press, 2018).
50. D. R. Schrider, J. Ayroles, D. R. Matute, A. D. Kern, Supervised machine learning reveals introgressed loci in the genomes of *Drosophila simulans* and *D. sechellia*. *PLoS genetics* **14**, e1007341 (2018).
51. L. Naduvilezhath, L. E. Rose, D. Metzler, Jaatha: a fast composite-likelihood approach to estimate demographic parameters. *Mol. Ecol.* **20**, 2709-2723 (2011).
52. K. Csilléry, O. François, M. G. B. Blum, abc: an R package for approximate Bayesian computation (ABC). *Methods Ecol. Evol.* **3**, 475-479 (2012).
53. L. Raynal *et al.*, ABC random forests for Bayesian parameter inference. http://dx.doi.org/http:// arXiv:1605.05537v5.
54. N. B. Edelman *et al.*, Genomic architecture and introgression shape a butterfly radiation. *Science* **366**, 594-599 (2019).
55. C. M. Jones *et al.*, Complete *Anopheles funestus* mitogenomes reveal an ancient history of mitochondrial lineages and their distribution in southern and central Africa. *Sci. Rep.* **8**, 9054 (2018).

56. L. L. Koekemoer *et al.*, Cryptic species within *Anopheles longipalpis* from southern Africa and phylogenetic comparison with members of the *An. funestus* group. *Bull. Entomol. Res.* **99**, 41-49 (2009).
57. R. J. Kent, M. Coetzee, S. Mharakurwa, D. E. Norris, Feeding and indoor resting behaviour of the mosquito *Anopheles longipalpis* in an area of hyperendemic malaria transmission in southern Zambia. *Med. Vet. Entomol.* **20**, 459-463 (2006).
58. A. Miles *et al.*, Genetic diversity of the African malaria vector *Anopheles gambiae*. *Nature* **552**, 96-100 (2017).
59. A. J. Geneva, C. A. Muirhead, S. B. Kingan, D. Garrigan, A new method to scan genomes for introgression in a secondary contact model. *PLoS One* **10**, e0118621 (2015).
